# Impact of ROS Generated by Chemical, Physical, and Plasma Techniques on Cancer Attenuation

**DOI:** 10.3390/cancers11071030

**Published:** 2019-07-22

**Authors:** Sarmistha Mitra, Linh Nhat Nguyen, Mahmuda Akter, Gyungsoon Park, Eun Ha Choi, Nagendra Kumar Kaushik

**Affiliations:** 1 Applied Plasma Medicine Center, Plasma Bioscience Research Center, Seoul 01897, Korea; 2 Department of Plasma Bio-display, Kwangwoon University, Seoul 01897, Korea; 3 Department of Electrical and Biological Physics, Kwangwoon University, Seoul 01897, Korea

**Keywords:** reactive oxygen species, reactive nitrogen species, free radicals, cancers

## Abstract

For the last few decades, while significant improvements have been achieved in cancer therapy, this family of diseases is still considered one of the deadliest threats to human health. Thus, there is an urgent need to find novel strategies in order to tackle this vital medical issue. One of the most pivotal causes of cancer initiation is the presence of reactive oxygen species (ROS) inside the body. Interestingly, on the other hand, high doses of ROS possess the capability to damage malignant cells. Moreover, several important intracellular mechanisms occur during the production of ROS. For these reasons, inducing ROS inside the biological system by utilizing external physical or chemical methods is a promising approach to inhibit the growth of cancer cells. Beside conventional technologies, cold atmospheric plasmas are now receiving much attention as an emerging therapeutic tool for cancer treatment due to their unique biophysical behavior, including the ability to generate considerable amounts of ROS. This review summarizes the important mechanisms of ROS generated by chemical, physical, and plasma approaches. We also emphasize the biological effects and cancer inhibition capabilities of ROS.

## 1. Introduction

Reactive oxygen species (ROS) are free radicals that are known to function as very important intracellular messengers [1] and can also modulate a wide range of mechanisms within the biological system, including various disease pathogenesis [2]. They are very well known for playing both beneficial and harmful roles in the human body [3,4]. Given this “double-edged sword” characteristic of ROS [5], especially with regard to the molecular mechanisms of cancer [6,7], it is very important to analyze and control the level of ROS required to instigate positive effects. From recent studies, it is established that ROS have the ability to play a crucial role in destroying cancer cells by means of enhanced oxidative stress through a variety of mechanisms [8,9,10,11]. As cancer pathology is related to a large number of receptors and molecular characteristics [12,13], changes in ROS levels can modify several pathways related to cancer. In addition, ROS can function as a treatment strategy for cancer if their level can be controlled within a beneficial range. Thus far, ROS have been targeted by a number of anticancer drugs, which act through various mechanisms to fight this disease [14]. Radiation is one of the most commonly applied cancer treatments that has the ability to produce ROS [15,16]. Nonetheless, we are not able to win the battle against cancer due to the present challenges of cancer therapy, such as drug resistance and the increasing side effects of conventional therapies. Moreover, the death rate caused by cancer is increasing [17]. Hence, in order to overcome this challenge, it is essential to introduce a noble therapy that can control the amounts of reactive species in the body. Eventually, changing the ROS amount can have anticancer effects both directly and indirectly with minimal side effects. In the quest for a new warrior in the present state of cancer treatments, it is found that treatments targeting ROS by any new tool can promise a new and more successful era of cancer therapy [14]. However, the most beneficial and preferable strategy would require the considerations of maintaining the optimum production and control of ROS to produce the effect not only on killing cancer cells, but also on inhibiting cell proliferation and the metastasis of cancer.

Plasma, the fourth state of matter, is generated by ionizing gas with high electrical energy. Plasma is categorized into thermal plasma and non-thermal plasma (or cold plasma) based on the nature of the electrons, ions, and neutral species. Particularly, non-thermal or cold plasma does not take place in the same local thermodynamic equilibrium state as thermal plasma, which results in the inequitable temperature between plasma species. The plasma electron temperature might reach up to tens of thousands Kelvin, far exceeding the temperature of the neutral gas, which remains around room temperature. Moreover, non-thermal plasmas devices such as plasma jet or dielectric barrier discharge (DBD)plasma normally operate in room condition, and are thus very suitable for life science research studies and applications [18,19]. The last two decades have witnessed a vast upswing of non-thermal plasma technology, from theoretical and experimental research, to real-life applications in various fields. A novel interdisciplinary field called “plasma medicine” has been created, concerning the generation of plasma at atmospheric pressure with room temperatures for treating living cells, DNA, and life science targets [20]. Non-thermal plasma produces various reactive species that can be used to enhance the oxidative stress of cancer cells and eventually kill cancer cells [21,22,23]. Diverse ROS can be generated in plasma, and some may increase the oxidative stress of cells [24]. As a result, it can modify any pathway that is directly or indirectly controlled by or related to ROS. It is currently established that the killing effect of plasma stems from the enhanced oxidative stress in cancer cells caused by plasma [22,25]. Recent works have shown that cancer cells produce more ROS [26,27], and therefore are prone to be affected by a rise in oxidative stress compared to normal cells, making them more suitable for being targeted by ROS [14] in conjunction with plasma technology. The killing effect of plasma is more prominent in cancer cells compared to normal cells, which makes the outcome of such a plasma treatment for cancer more fruitful.

Despite being a new method, plasma has already been used in different fields of medicine and surgery [28,29,30]. It has been also applied successfully in the fields of dentistry [31], sterilization [32], and skin treatment [33]. Evidence from recent works suggests that most of the activity of plasma comes from the production of reactive species. As a result, it is considered as a new tool in the field of oncology, and researchers have started to explore the effects of plasma on carcinoma cases. Plasma has been applied in cancers of various organs such as breast [34], ovarian [35], prostate [36], lung [37], brain [38], and skin [39], with results showing the excellent effects on these types of cancer. Although it has not been applied clinically to cancer patients, in vitro and in vivo experiments testify that plasma technology has great potential to be used as a therapeutic treatment for cancer in the future [40]. It remains not well known how plasma generates ROS and/or if there are any other clear mechanisms related to the killing of cancer cells. Moreover, there are various types of plasma devices, and plasma can be produced using a large variety of gases or combinations of gases. All of these aspects are very influential on the physical characteristics of plasma. Hence, it is not certain that all in vivo and in vitro experiments done thus far can be applied under identical conditions or can be compared directly.

The present review attempts to combine the intracellular production of ROS and the generation of ROS by different physical and chemical means. Furthermore, we aim to discern the underlying mechanisms of ROS with reference to cancer inhibition and the potential of plasma to be developed as a future cancer treatment technology.

## 2. Types of Reactive Oxygen Species

The ground state of diatomic oxygen is called triplet oxygen (^3^O_2_), containing two unpaired electrons with parallel spins in the outer valence shell. Due to selection rules, the oxidation reaction of triplet oxygen can occur with organic molecules that donate an electron pair with parallel spins. Thus, the reactivity of triplet oxygen is relatively low since electron pairs of organic molecules typically have anti-parallel spins. Nevertheless, ^3^O_2_ can be converted into several highly reactive molecules, specifically ROS, via either electron-transfer or energy-transfer processes. The term ROS is used to describe several radical and non-radical molecules that derive from diatomic oxygen. The four most important ROS in a biological system are the superoxide anion, singlet oxygen, hydrogen peroxide, and hydroxyl radicals.

### 2.1. Singlet Oxygen

Singlet oxygen (^1^O_2_) is the lowest electronically excited state of diatomic oxygen. ^1^O_2_ is a highly reactive molecule that can inflict severe damage on cells. It is also involved in the signaling of apoptosis and acclimation processes. ^1^O_2_ is the only ROS generated by energy transfer, whereas the other types are products of electron-transfer reactions. In general, ^1^O_2_ is indirectly formed via a photosensitization process during which a photosensitizer is excited by irradiation, followed by energy transfer to excite the ground-state triplet oxygen into the singlet oxygen state [41]. Singlet oxygen is highly unstable and constantly decays back to the ground state, emitting radiation in the near-IR region. These reactive properties make singlet oxygen an important factor in photodynamic therapies. In addition, ^1^O_2_ plays a role in a variety of chemical reactions to produce other oxidants and other highly reactive and cytotoxic molecules, which can directly inactivate antigens and kill pathogens [42].

### 2.2. Superoxide Anion ^•^O_2_^−^

Superoxide anions (^•^O_2_^−^) are a product of the one-electron reduction of diatomic oxygen and are the most common type of ROS [43]. Under physiological conditions, ^•^O_2_^−^ can be generated by the NADPH oxidase enzyme or by the mitochondrial electron transport chain [44]. The reactivity of the ^•^O_2_^−^ molecule is widely considered to be relatively low. ^•^O_2_^−^ can be dismutated into two less reactive species, oxygen and hydroperoxide, in the presence of an enzyme called superoxide dismutase (SOD). On the other hand, ^•^O_2_^−^ participates in the Haber–Weiss reaction, generating hydroxyl radicals, which are the most reactive and most dangerous type of ROS [45]. This reaction is a cause of oxidative stress in cells. Thus, ^•^O_2_^−^ is still capable of indirectly inflicting biological damage.

### 2.3. Hydroxyl Radicals

Hydroxyl radicals (^•^OH) are an extremely reactive oxidizing species and are the most dangerous ROS, with a strong tendency to react with other molecules due to the presence of an unpaired electron [46]. In general, ^•^OH forms as a result of the dismutation of ^•^O_2_^−^ in the presence of an excess of superoxide anions with metal ions as a catalyst (Haber–Weiss reaction). Owing to its strong instability, ^•^OH is capable of interacting with all types of biological molecules [47]. Several forms of severe damage to cellular components, such as lipid peroxidation, damage to proteins, and membrane destruction can be caused by ^•^OH. Nevertheless, cells have no enzymatic mechanism to eradicate ^•^OH; thus, an excessive concentration of ^•^OH can result in cell death. 

### 2.4. Hydrogen Peroxide

Hydrogen peroxide (H_2_O_2_) is a type of ROS with a relatively long lifetime. H_2_O_2_ molecules can take part in reactions with other molecules at sites distant from where they are produced as they are permeable to biomembranes, which is most likely via the aquaporins of cell membranes. Although not a radical, H_2_O_2_ is capable of reacting with transition-metal ions to form ^•^OH [41]. H_2_O_2_ has high potential to cross membranes, and thus can be used as a second messenger for signaling cascades using ROS [48,49].

## 3. Generation of ROS

**Figure 1:**
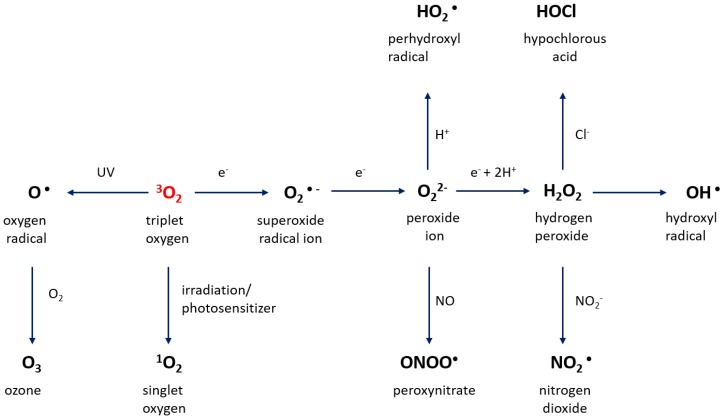
Schematic of primary reactive oxygen species (ROS) production mechanism.

Reactive oxygen species are generally produced as by-products of different types of aerobic metabolism [50]. It is well known that ground-state triplet molecular oxygen is a bio-radical containing two valence electrons with parallel spins occupying separate orbitals [51]. Generally, triplet oxygen interacts with an agent to provide a pair of electrons with parallel spins, which may enter two different electron orbitals and eventually oxidize a non-radical atom or molecule [52]. Nevertheless, these pairs of electrons in most cases have opposite spins. Therefore, this may create restrictions in reaction cases with triplet molecular oxygen with most organic molecules [53,54]. As a result of an energy transfer or by means of electron transfer reactions, ground-state oxygen may be changed to a much more reactive type of ROS, leading to the production of the singlet oxygen or superoxide, hydrogen peroxide, and hydroxyl radicals [55]. ROS can be produced in a living system by a variety of processes, such as biological metabolism, enzymatic processes, and as by-products of biological reactions. Moreover, various types of chemical reactions can be responsible for the production of ROS. The prominent pathways of ROS production are illustrated in Figure 1.

### 3.1. Intracellular Production of ROS

**Figure 2:**
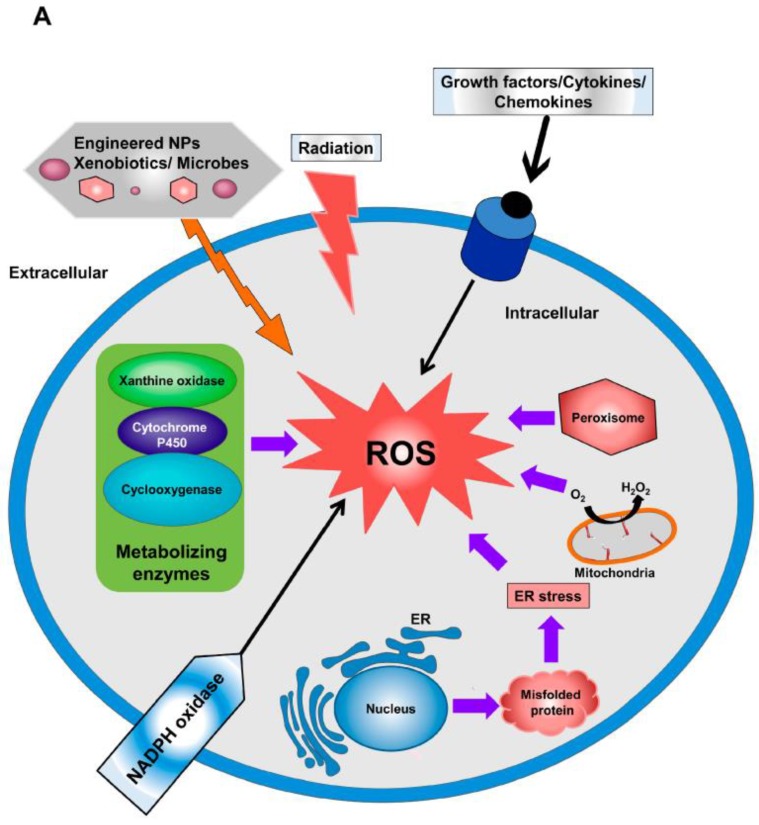
Some major intracellular (mitochondria, peroxisome, endoplasmic reticulum (ER) stress, nicotinamide adenine dinucleotide phosphate hydrogen (NADPH) oxidase, metabolizing enzymes) and extracellular (Radiations, Xenobiotics) sources of reactive oxygen species (ROS) generation [81].

A vast number of different biological processes generate ROS, and the sources and production of ROS have been studied by numerous researchers. Figure 2 demonstrates the primary intercellular and extracellular ROS generation mechanism. ROS can be produced by the induction of various cytochrome P_450_ isoenzymes during the detoxification of chemical carcinogens. Again, lipid peroxidation and other intracellular processes involved in NFkB, transcription factors kappa B and AP-1—partly by means of protein kinase C activation [56,57] and PPARγ leading to chronic inflammation [58]—can cause the intracellular generation of ROS. During the primary response to oxidative stress, transcription factor NF-E2-related factor 2 (Nrf2) regulates a large number of antioxidants and cellular protective genes [59]. Moreover, numerous factors can be related to the production of ROS. Most ROS produced intracellularly originate in the mitochondrial respiratory chain and subsequently create by-products that are toxic in nature. Mitochondria, being the most important character in this play, represents the main intrinsic source of ROS generation using mitochondrial ETS (electron transport system) [60]. ROS generated via the ETS of mitochondria are known to be involved in the pathways of cellular signaling, which are related to survival and cell death [61]. During these processes, the secretion of mitochondrial ROS to the cytosol is strictly modulated by a large group of proteins [62]. This ROS release is implicated in redox homeostasis control and in a large variety of cellular signaling pathways. Oxidative ATP production is one of the main functions of mitochondria [63], during which water is produced by the reduction of oxygen (O_2_). Consequently, the mitochondrial respiratory chain is one of the most important and major sources of intracellular ROS generation [64]. At the time of respiration, electrons released from the mitochondrial electron transport chain incompletely reduce O_2_ to form superoxide [65]. By the action of manganese superoxide dismutase (Mn-SOD), superoxide is transformed into H_2_O_2_ in the mitochondrial matrix or by Cu and Zn-SOD in the intermembrane space (IMS) of mitochondria [66,67,68]. The 1–2% of O_2_ consumed during respiration is estimated to be completely reduced to O_2_ in order to generate superoxide in isolated mitochondria treated with respiratory chain inhibitors [65]. However, 0.12–0.15% of O_2_ can generate H_2_O_2_ when palmitoyl-coenzyme A or glutamate/malate serve as the substrate [69]. Recently, it was reported that mitochondrial superoxide is generally formed at seven major sites of mitochondria, and that all sites eventually release it into the matrix [70]. The two major sites of the generation of superoxide are complexes I and III, which are parts of the mitochondrial respiratory chain. It was also reported that the increased accumulation of calcium (Ca^2+^) in the cytoplasm is responsible for activating the mitochondrial electron transport chain and for the production of ROS. Another major endogenous source of ROS is the mammalian cytochrome P450 (CYP)-dependent microsomal electron transport system [71].

The endoplasmic reticulum (ER) plays a key role in ROS production that is related to ER stress. Previous works suggest that any change in redox homeostasis in the ER can be responsible for ER stress, which as a result can enhance the production of ROS in mitochondria and in the ER [72]. In the ER lumen, secretory proteins and the correct folding of most membranes demand the formation of disulfide bonds between cysteine residues, which is a reversible process, in order to stabilize tertiary as well as quaternary structures [73]. Several ER oxidoreductases, protein disulfide isomerases (PDI), ERp72, and ERp57 are involved in oxidative protein folding in eukaryotic cells. Additionally, this protein folding process is thermodynamically as well as kinetically controlled by the redox state of the microenvironment, and maintained by redox buffers of the lumen of ER, including thiol–disulfide pairs and reduced or oxidized pyridine nucleotides [74]. Glutathione (GSH) is one of the most significant and abundant thiols in eukaryotic cells, and it can be converted into glutathione disulfide by oxidation (GSSG) [10]. This explains why redox homeostasis in the cell is maintained by the balance between GSH and GSSG [75]. In the cytosol, a ratio of GSH/GSSG ranging from 30:1 to 100:1 creates a reducing environment, while in the lumen of the ER, the GSH/GSSG ratio is as high as 1:1–3:1, representing an oxidized environment [76]. This oxidized environment in the ER lumen is essential for oxidative protein folding. Additional sources include cardiac and vascular cells [77]; brain cells [78]; phagocytic cells such as leukocytes, macrophages, monocytes, neutrophils, and eosinophils; and various oxidases such as nicotinamide adenine dinucleotide phosphate hydrogen (NADPH) oxidases, aldehyde oxidase, glucose oxidase, and xanthine oxidase. Neutrophils, eosinophils, and macrophages are supplementary endogenous sources and are very significant contributors to the production of ROS. Activated macrophages can cause a “respiratory burst”, showing a rapid but transient enhancement in oxygen uptake that is responsible for higher production levels of superoxide anions, hydrogen peroxide radicals, and a variety of other ROS [79].

Peroxisomes are another important cellular source of production of ROS. Oxygen is consumed by these cellular organelles, which in turn generate hydrogen peroxide and superoxide. ROS generation also includes a battery of peroxisomal oxidases together with acyl-CoA oxidase and xanthine oxidase, creating hydrogen peroxide and superoxide. The amount of oxidases and H_2_O_2_ produced differ among cells and tissues [80].

### 3.2. Roles of Different Enzyme and Protein Expression Levels during the Intracellular Production of ROS

Cytochrome oxidase is a large membrane-associated multiprotein assembly containing transition metal ions (iron and copper) at its active sites and allowing the transfer of single electrons to facilitate redox reactions [82]. The terminal cytochrome oxidase complex catalyzes several single-electron reduction steps, by which four electrons are added sequentially to each O_2_ molecule at normal oxygen levels. Cytochrome oxidase keeps the partially reduced oxygen intermediates formed during the reduction process safely bound until they can be fully reduced to water, without releasing superoxide radicals or other reactive oxygen radicals from the mitochondrial membrane [83].

Cytochrome P450 enzymes function as part of the NADPH/O_2_-dependent microsomal electron transport system, and are one of the major cellular sources of ROS [71,83,84,85,86]. The ability of CYP enzymes to incorporate an oxygen atom from O_2_ into numerous organic substrates (monooxygenase activity), to utilize H_2_O_2_ and cumene hydroperoxides well as other hydroperoxides as oxygen atom donors to oxygenate substrates (peroxygenase activity), and to use H_2_O_2_ and other peroxides during the one-electron oxidation of substrates (peroxidase activity) demonstrates the catalytic versatility of CYP enzymes [85]. During catalysis by microsomal CYP enzymes, two electrons are acquired from NADPH and migrate from the flavin adenine dinucleotide domain of the flavoproteins reductase to the CYP heme group. A water molecule normally occupies the sixth coordination site of heme iron, but is replaced by molecular oxygen when the catalytic reaction begins [85]. The CYP monooxygenase cycle commences with the iron of the heme group in the oxidized ferric state (FeIII) (A) and continues until ROS such as O_2_, H_2_O_2_, and ^•^OH are eventually liberated as opposed to a monooxygenerated substrate in a process known as uncoupling [85,87].

### 3.3. Generation of ROS by Chemicals

The generation of oxidative stress and damage has been found to occur following exposure to xenobiotics with various chemical structures and modes of action. Chlorinated compounds [88], barbiturates [89], phorbol esters [90], and certain peroxisome proliferating compounds [80] are among the classes of compounds involved in induced oxidative stress and damage via both in vitro and in vivo methods [91]. 2-Butoxyethanol is a chemical that can generate ROS by an indirect mechanism [92]. The production of ROS can be induced by the chemical 8-hydroxyguanosine in the liver, which is produced by the activation of Kupffer cells secondary to 2-butoxyethanol-induced hemolysis as well as subsequent hepatic iron deposition [93]. A number of other compounds, such as dieldrin [94], 2,3,7,8-Tetrachlorodibenzo-*p*-dioxin [95], lindane [96], and phenobarbital [97] have been proved to function as a source of reactive species in the human body [98].

The superoxide-driven Fenton reaction plays a major role in converting poorly reactive radicals into highly reactive ones [99,100]. The Fenton reaction is defined as the reaction of ferrous iron (Fe^2+^) and hydrogen peroxide (H_2_O_2_). In this reaction, ferric iron (Fe^3+^) and ^•^OH are produced. Then, A ^•^OH reacts with H_2_O_2_, and superoxide (O_2_^−^) is produced [101]. Then, the superoxide reacts again with H_2_O_2_, forming an ^•^OH and a hydroxyl anion (^−^OH); this part of the reaction is known as the ‘Haber–Weiss Reaction’ [102]. Superoxide (O_2_^−^) is reduced to Fe^3+^ rather than H_2_O_2_. Several metals such as Fe, Cu, Zn, and Al have oxygen-transferring properties, giving them the catalytic power to generate highly reactive ^•^OH by the Fenton reaction [101]. ^•^OH is mainly involved in three types of reactions: hydrogen abstraction, the addition reaction, and the oxidation reaction [103].

Fe ^2+^ + H_2_O_2_ = Fe^3+^ + ^•^OH + ^−^OH [Fenton reaction] 

^•^OH + H_2_O_2_ = ^•^O_2_^−^ + H^+^ + H_2_O

O_2_^−^ + H_2_O_2_ = ^•^OH + ^−^OH + O_2_ [Haber–Weiss reaction]

### 3.4. Generation of ROS by Radiation

A very well-known and widely evaluated source of ROS is radiation energy [104]. The radiation of various types and ranges causes the generation of different type of oxygen species. UV-B light in the range of 1–100 mJ/cm^2^ causes a distinct rise in the generation of ROS in human and mouse keratinocytes cells [105]. The production of ROS depends on the dose of the UV-B light. Depleting the keratinocytes of glutathione using an inhibitor of glutathione synthesis increases the level of intracellular ROS [106]. Moreover, glutathione-depleted cells were considerably more responsive to the oxidant-generating nature of UV-B light [106].

The ionizing radiation (IR) is electromagnetic radiation, which has the ability to remove electrons from atoms. The most commonly used types of radiation for the treatment of cancer are X-rays, gamma rays, and charged particles [107]. IR is initially responsible for the ionization and excitation of water, eventually causing the formation radiolysis products from water, such as hydrated electrons, ionized water, hydroperoxyl radicals (HO_2_^•^), ^•^OH, hydrogen radicals (H^•^), and H_2_O_2_ in a very short span of time (10^−8^ s) when irradiated in a biological system [104]. This eventually creates a side effect of low linear energy transfer (LET) IRs such as γ-rays and X-rays [108]. It is also evident that when irradiated onto cells, IR not only causes the generation of ROS from water radiolysis; it also has been found that IR is responsible for enhancing the intracellular level of ROS, including O_2_^−^ several hours after exposure [15,109,110]. It was found and established by Yamamori et al. that the IR-induced G2/M arrest led to a sustained increase in cells with enhanced mitochondrial quantities and higher levels of cellular oxidative stress, thereby causing an increase in the oxidative stress in all the cells after their exposure to radiation [111]. 

A laser is a source of light or radiation energy. The low-level laser (LLL) is a specific type of laser that has the ability to affect biologic systems without causing an increase in the temperature [112]. According to Karu, exposure to laser irradiation results in an increase in mitochondrial electrochemical activity and a concomitant enhancement in ATP synthesis [113]. It has also been reported that cytochrome c oxidase is the main photoreceptor of laser light [114,115]. Additionally, the low-level laser has a cascade effect on cell signaling, which plays a role in cellular proliferation and cytoprotection [116]. In some studies, it was also reported that laser therapy influences oxidative stress parameters, for instance changing the level of antioxidant enzyme activity and generating ROS [117,118,119]. The absorption of laser light boosts the transfer of electrons in the respiratory chain, causing a sudden increase in the initial level of ROS production, specifically enhancing the generation of superoxide anions. However, the role of laser irradiation on the cellular mechanism and its effect on oxidative parameters are still not clearly known [120].

### 3.5. ROS Production by Plasma

Atmospheric pressure plasmas are very well known for creating very high concentrations of various types of reactive species. It was reported that DBD plasma and jet plasma can generate large amounts of ROS [22,121,122]. Indirect plasmas are generated between two electrodes of certain devices and are transported to the application area via a gas flow. ROS are usually generated at the boundary of the jet with the adjacent air by a number of different mechanisms [121]. According to several authors, ROS produced by plasma can cause morphological changes, the depolarization of membranes, lipid peroxidation, and damage to DNA in cells [123,124]. The anti-neoplastic activity of CAP is primarily based on the delivery of reactive oxygen and nitrogen species (RONS) [24]. For plasma medicine, the determination of the amount of reactive species produced in plasma-treated liquids is of enormous value. Currently, numerous lines of research are focused on applying plasma as a cancer treatment using the ROS production property [125,126,127,128]. Treatment with plasma causes the depolarization of the mitochondrial membrane potential and results in the formation of ROS in human cells [129]. It has been reported that the therapeutic effects of air plasma result from the production of RONS such as H_2_O_2_, O_x_, OH^−^, ^•^O_2_^−^, and NO_x_ due to the depolarization of the mitochondrial membrane potential and mitochondrial ROS accumulation [126].

### 3.6. ROS Production by Anticancer Drugs during Cancer Therapy

A number of studies demonstrate that anticancer drugs can cause oxidative stress in cancer patients treated with chemotherapy [130]. However, there is a very significant association between enhanced oxidative stress and the effects of natural anticancer agents such as sesquiterpene lactone parthenolide [131]. A noble phenolic compound derived from hispidin has been reported to act against colon cancer by generating ROS and causing apoptosis by both intrinsic and extrinsic pathways [132]. In cancer cells, ROS signaling is a key factor playing important roles in a number of stages, such as survival, transcription, protein translation, and tumor formation and development. The ROS hydrogen peroxide results in the apoptosis of cancer cells [133], and a number of anticancer drugs can produce this agent to show an anticancer effect [134,135]. Data from a recent study by Yokoyama et al. suggest that nimustine, actinomycin D, doxorubicin, mitomycin C, mitoxantrone, carmofur, gemcitabine, mercaptopurine, camptothecin, paclitaxel, vinblastine, and vinorelbine can cause significant oxidative stress [136]. Vinorelbine, an anticancer agent, depletes intracellular GSH and increases intracellular ROS production [137]. Enhanced levels of oxidants in the blood circulation have been found in patients with cancer after an administration of epirubicin [138]. Several anticancer drugs initiate DNA damage and result in subsequent apoptosis induction. Epirubicin [139] and doxorubicin [140] can generate ROS, causing damage to the DNA and eventually resulting in antitumor activity [141]. TAS-103 also shows anticancer action by oxidative DNA damage [142]. Eriocalyxin B [143], artemisinin [144], genipin [145], gemcitabine [146], spiclomazine [147], belinostat [148], artesunate [149], isoalantolactone [150], and dihydro artemisinin [151] have been found to cause an enhancement of ROS by various mechanisms, eventually inhibiting cancer proliferation via ROS-mediated mechanisms. The most well-known anticancer drugs for producing ROS and their mechanisms are summarized in Table 1.

## 4. ROS Roles in Cellular Mechanisms for the Inhibition of Cancers

Currently, cancer is one of the most lethal diseases worldwide, and it is now a great challenge to establish highly potent treatments for cancer by discovering new targets. The conventional approaches for treating cancer are not very effective in many cases due to multidrug resistance and the side effects of chemotherapy. As a result, many studies have been designed to find potential targets for cancer therapies. Numerous factors are highly significant and closely related to cancer initiation and development strategies.

Reactive oxygen species can also be a very important factor in cancer cases given the function of ROS as secondary messengers and considering the very close relationship with a number of cellular mechanisms, including those related to the survival of cells. Free radicals, mainly ROS, have been reported as very common mediators of apoptosis. Again, it has already been reported that certain chemotherapeutic agents and radiation therapies cause oxidative stress by enhancing ROS in patients when used as a cancer therapy. When the amounts of ROS rise to the toxic threshold level, the antioxidant system of the cell is eventually altered, possibly leading to cell death. In this scenario, the death of cancer cells can be increased by using exogenous ROS-generating agents, because they cause enhanced ROS stress. Oxidative stress can induce many biological responses, which may include a transient arrest of growth and adaptation, the initiation of signal transduction pathways, gene transcription, and damaged DNA repair [166,167]. These events determine whether a cell will undergo necrosis, senescence, apoptosis, or will survive and proliferate [167]. The extent of these responses can depend on the cellular genetic background, the different classes of specific ROS involved, and significantly on the intensity and duration of the oxidative stress created [168,169].

**Figure 3:**
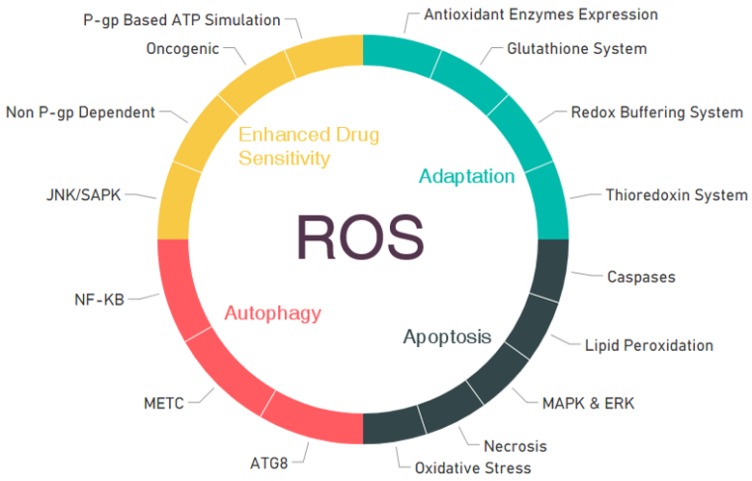
Role of reactive oxygen species (ROS) in cancer inhibition by four different mechanisms and the different pathways involved in those mechanisms.

Increased ROS in cells using a therapeutic approach can have anticancer effects by a number of different mechanisms. The four most important mechanisms—adaptation, apoptosis, autophagy, and enhanced drug sensitivity—are represented in Figure 3. This chapter focuses on the principle and impact of these above-mentioned mechanisms.

### 4.1. Adaptation

Reactive oxygen species homeostasis is necessary for cell survival, because high levels of ROS have toxic effects on cells, which initiates a signal transduction mechanism involving cell proliferation inhibition or cell death [170]. It is important to note that the amounts of ROS in cancer cells are higher than those in normal cells [171]. When low levels of ROS stress are induced in cells, the cells become able to regulate various types of adaptation mechanisms and make adjustments given the increased level of oxidative stress [61]. In order to equilibrate the increased ROS effect, ROS can cause redox buffering systems [172] and various antioxidant enzymes [61,173] to be generated by cells. The glutathione system (GSSG/2GSH) [174] and the thioredoxin system [175] are the most abundant redox couples involved in maintaining the cellular redox balance to detoxify the effect of certain types of ROS. The mobilization of redox-buffering systems can be considered as the first instance of cellular adaptation to ROS stress [176]. The upregulation of antioxidant enzyme expression levels of, for instance, SOD, catalase, and peroxidase represents a very significant adaptation mechanism, providing more sustainable protection against increased ROS stress. However, such adaptation processes are inadequate for killing cancer cells. Nonetheless, under sustained ROS stress conditions, adaptation mechanisms and the weakening of the ROS-buffering capacity are both highly likely. Anticancer chemotherapeutic agents can produce exogenous ROS, eventually leading to ROS stress such that it activates cell death [177].

Adaptation developed by ROS can play a very important role in cancer treatments by several different types of pathways, but a single and specific mechanism that can be more promising and can act more selectively compared to others has yet to be found. Generally, this process works with other apoptotic mechanisms to kill cancer cells, and the combination can eventually enhance the rate of apoptosis. Future works should focus on finding appropriate amounts or concentrations of ROS to initiate the adaptation process in order to design new therapeutic approaches.

### 4.2. Apoptosis

Chemotherapy, radiotherapy, and other therapeutics involved in cancer treatments in most cases can produce ROS, and these approaches mostly target mechanisms that kill cells. Several mechanisms to explain apoptosis initiated by ROS have been considered. Indeed, the excessive production of ROS in cells is known to induce apoptosis [177,178,179]. The excessive generation of ROS may cause damage to cellular components, including the DNA, proteins, and lipid membranes [180]. Protein damage can be caused by direct oxidative alteration of the side chains of amino acids and by ROS-mediated peptide cleavage [181]. The oxidation of proteins can demolish the redox equilibrium, which is essential for ensuring the appropriate roles of numerous metal-containing enzymes, including cytochrome c, cytochrome c oxidase, glutathione peroxidase, and catalases. The inhibition of catalases and peroxidases by oxidation sequentially decreases the ability of cells to eliminate H_2_O_2_ and further escalates oxidative stress.

The nitrosylation of protein is another mechanism by which ROS cause cellular injury and apoptosis. Peroxynitrite, the product of a reaction between superoxide and nitric oxide, is a major ROS that causes the nitrosylation of proteins. It also influences the roles of signaling molecules such as NF-kB, AP-1, and p53 [182].

Several apoptosis-related signaling pathways, such as the MAPK (mitogen-activated protein kinase) pathway and the ERK (extracellular signal-regulated kinase) pathway, are reportedly involved in ROS-induced apoptotic cell death [183]. Apoptosis caused by death receptors and mitochondria depends on ROS levels in the cells, resulting in oxidative stress [184]. The Fas ligand (FasL) activates fast ROS generation, which is mostly derived from NADPH oxidase, an earlier event of Fas stimulation and the starting point of apoptosis. p38, another member of the MAPK family, is also involved in apoptotic signaling as a result of the increased generation of ROS. p38 and JNK (c-Jun N-terminal kinase) are both activated by Ask-1 (apoptosis signal-regulating kinase-1), whose action is controlled by its interaction with thioredoxin, another redox-regulated protein [185,186]. In addition, Ask-1-induced signaling cascades and certain other signaling proteins such as FOXO3a, p66Shc, and p53 are involved in apoptosis initiation in response to ROS [187,188].

Reactive oxygen species play important roles in initiating apoptosis processes by affecting various signaling cascades and by directly oxidizing cellular proteins, lipids, or nucleic acids and causing general damage and dysfunction. ROS can also affect various crucial necrotic pathways that can also lead to a certain amount of necrotic cell death [189], which is a faster and less energy-dependent event compared to apoptosis. Again, death receptors, for instance, TNF (tumor necrosis factor) receptor-I, enhance ROS generation via the mitochondria, leading to the activation of caspases and causing cell death [190]. However, TRAF4 (TNF receptor-associated factor 4), which is a factor of the TNFα signaling pathway, binds to the NADPH oxidase complex in order to trigger JNK signaling [191], which suggests that death receptors use several pathways to induce ROS within cells. Notably, TNF-induced oxidative stress also activates anti-apoptotic signaling by increasing the expression levels of MnSOD and catalase by NF-Κb [192].

Lipid membranes are vulnerable to ROS attack. Once lipid peroxidation is initiated, it produces organic radicals, which consecutively initiate the proliferation of peroxidation reactions and cause substantial damage [193]. Lipid peroxidation can reduce the fluidity of biological membranes and enhance the permeability of these membranes [194]. Since much of the ^•^O_2_^−^ is generated in the mitochondria, damage to the mitochondrial membrane is likely to be the cause of the release of cytochrome c, stimulating the cascade of apoptosis. Mitochondrial membrane potential reduction, the destruction of the mitochondrial respiratory chain, and ATP depletion are general consequences of enhanced oxidative stress [195,196]. Cytochrome c leakage from the permeability transition (PT) pore complex, apoptosome production, and the triggering of caspases are the most important measures of mitochondrial-induced apoptosis.

A recent study showed that peripheral T cells cultured in the absence of survival factors may gather ROS, upregulate the expression levels of the Bcl-2-interacting mediator of death (BIM) and inducible nitric oxide synthase (iNOS), and undergo apoptosis, which is inhibited by antioxidants [197]. However, the enormous cellular oxidation caused by elevated levels of ROS may bring about the death of narcotic cells rather than apoptosis [196]. It is possible for ROS to prompt either of these death responses, and apoptosis and necrosis may occur together in the same tissue [198]. The power of ROS to impose severe cellular damage together with cell death provide a chance to destroy cancer cells by excessive ROS stress imparted to malignant cells by means of pharmacological agents.

It is also very important that a significant pathway shared in general by chemotherapy and radiotherapy is ROS-induced apoptosis [199]. Most of the recently developed anticancer drugs, such as Levistolide A [200], TAS-103 [142], and doxorubicin [142] have been reported to provoke apoptotic cell death in tumor cells by the generation of ROS [201]. Studies have shown that ROS can cause apoptosis by enhancing the activity of caspases and eventually the overexpression of death receptor 5 (DR5). The protein kinase C (PKC) zeta-reliant phosphorylation of p47-phox confers NADPH oxidase activation. The FasL-activated ROS response is crucial for the interaction between epidermal growth factor receptor (EGFR) and Fas as a sign of its phosphorylation. Moreover, tyrosine leads to the initiation of apoptosis by recruiting the Fas-linked death domain and caspase-8 [202,203]. In addition, FasL-promoted ROS production aids with ubiquitination followed by the inhibition of the function of the caspase-8/FADD-like IL-1beta-converting enzyme (FLICE) inhibitory protein (FLIP) to assist with the activation of Fas [204]. ROS affect the structural integrity of the PT pore by signaling cascades and through the oxidative modification of the PT pore structure. The JNK signaling pathway is initiated by ROS, activating apoptosis signal-regulating kinase 1 (ASK1) by releasing mitogen-activated protein kinase kinase 1 (MEKK1) from its attachment with glutathione S-transferase (GST) [205] or by blocking the action of Protein tyrosine phosphatase (PTP) to allow for the functioning of Srctoinitiate downstream signaling [206].

In summary, increased ROS can encourage apoptosis in cancer cells by a variety of mechanisms, and this aspect can be therapeutically implicated via a ROS-boosting anticancer therapy. Many well-established anticancer agents are already known to show action in this way. Hence, more research should be designed to find which types of cancer are more susceptible to ROS-boosting treatment strategies, as different cancers may show different characteristics and act differently when treated with ROS-generating agents. Again, maintaining the balance of ROS in cells is also a very important factor when developing this type of therapeutic strategy, as ROS can also play a positive role in metastasis. Currently, researchers are considering both ROS-enhancing and ROS-depleting treatment strategies on the basis of the type of cancer. Therefore, future works should focus on discovering the roles of ROS-boosting cell-killing strategies for specific cancers that show better results than other methods and finding strategies that maintain the balance of ROS during the treatment. This is important because ROS can also show toxic effects if there is a major misbalance in the approach, and in the long run can worsen the situation. Thus far, ROS-based cancer treatments have shown remarkable progress, which makes the situation more challenging to those seeking solutions with regard to the main obstacles of this strategy.

### 4.3. Autophagy

Autophagy can simply be defined as the process of the degradation of proteins and organelles, which may recycle in order to form new cells. It plays a key role in cellular reactions as a response to increased ROS levels. It is a multi-step operation that controls cellular homeostasis by degrading and recycling long-lived proteins and intracellular aggregates together with damaged organelles. This process requires nearly 40 proteins and can demonstrate the formation of a double-membrane structured phagophore that engulfs part of the cytoplasm and organelles in order to create an autophagosome. The initiation of autophagy is synchronized by two kinases, unc-51-like kinase 1 (ULK1) and vacuolar protein sorting-34 (VPS34). The adenosine monophosphate (AMP)-dependent protein kinase is a key factor in controlling ULK1 and mTORC1, which is based on the energy condition of the cell [207].

Recently, it was revealed that ROS can result in autophagy by various distinct methods involving Atg4, catalase, and the mitochondrial electron transport chain (mETC). This may cause both cell survival and cell death, but the action could selective toward cancer cells [8]. Accordingly, it is obvious from a number of research outcomes that in the case of survival-prone autophagy, ROS can function as an efficient signaling molecule [208]. It was reported by Poillet-Perez et al. that certain levels of ROS production regulate the induction of autophagy in cancer cells [209]. By causing the oxidation of enzyme ATG4 to ATG8 protein by H_2_O_2_, ROS can play a very significant role as a prerequisite for the induction of autophagy. This oxidation converts active ATG4 to an inactive form, resulting in the enhanced production of LC3-associated autophagosomes [209]. Indirectly, the adenosine monophosphate-activated protein kinase (AMPK) pathway is another significant factor related to the maintenance of autophagy by ROS [210]. AMPK activation can increase autophagy by restraining the mammalian target of rapamycin complex 1 (mTORC1). Oxidative stress can alter the AMPK pathway and initiate it by phosphorylating the AMPK kinase (AMPKK) and subsequently can increase the production of H_2_O_2_, which induces apoptosis indirectly [209]. ROS can also play a key role in autophagy by affecting the activity of various transcription factors such as NFκB, which is responsible for the expression of autophagy-associated genes in tumors [211]. Selenite causes cytotoxicity mediated by autophagy in human glioma tumors, and the excessive generation of the SOD enzyme conspicuously hinders autophagy stemming from selenite. siRNA helps in case of the knockout of autophagy-related gene 6 (ATG6) or ATG7, and reduces selenite-promoted autophagy. The application of ROS-derived autophagy in treating cancers has recently started [212,213]. In light of these results, it can be said that the enhanced generation of ROS and related treatment strategies can induce autophagy in cancer cells. At present, the challenge is to find the most effective and clear mechanisms of action played by ROS in autophagy, and doing so necessitates more work to establish safe and sound therapeutic applications.

### 4.4. Increased Action and Sensitivity of Anticancer Agents by ROS

From a number of research works, it has been found that anticancer agents produce ROS, which may eventually enhance the oxidative stress to a level that pushes it beyond the maximum tolerance level, ultimately causing death to cells [214]. Apart from being involved in direct damage to cellular molecules, ROS appears to play a unique role in controlling the apoptosis process, which is initiated by a range of anticancer therapeutic agents and other stimuli. A very common mechanism of various ROS-producing anticancer agents is a sudden increase of the ROS level within the cells, or a transient ROS burst [179,215,216]. This increased ROS generation in cancer cells makes the cells highly dependent on antioxidant enzymes to withstand ROS stress. The sustained oxidative stress due to the presence of constant oncogenic signals and active metabolism likely requires the full utilization of the cellular antioxidant capacity. In such cases, cancer cells with increased endogenous ROS stress levels should be more sensitive to anticancer agents that either cause further ROS generation or impair the cellular ability to eliminate ROS. Indeed, it has been observed that human leukemia cells with high ROS contents are more sensitive than normal lymphocytes (low cellular ROS) to 2-methoxyestradiol (2-ME), which is a novel anticancer agent that causes ROS accumulation by inhibiting SOD [217]. In an earlier section, it was discussed how ROS level increases are also associated with the initiation of the redox-sensitive JNK/SAPK (c-Jun N-terminal kinase /stress-activated protein kinases) signaling mechanism, which is generally engaged during the transcriptional activation of genes and during post-translational alterations of proteins required for apoptosis. In 2006, Kim et al. reported that the Bcl-2 Homology 3 (BH3)-only protein Noxa responds directly to hypoxia-inducible factor-1 (HIF-1) and seems to play an important role in hypoxia-induced cell death with the participation of ROS [218]. From the previous discussion, we found that ROS generation during the process of apoptosis is considered to be correlated with the malfunction of the mitochondrial respiratory chain, the disengagement of cytochrome c, and modification of the mitochondrial transmembrane potential and membrane permeability [219,220]. Although mtDNA and the respiratory function are not always essential for the process of apoptosis, their absence or the impairments of their functions can influence the rate of ROS generation and the kinetics of the apoptotic process and therefore modulate drug-induced apoptosis, possibly leading to the enhanced action of anticancer drugs [221].

Based on research conducted thus far, it has also been found that ROS can be involved in collateral sensitivity by means of either P-glycoprotein (P-gp)-based ATPase stimulation or non-P-gp-dependent ROS hypersensitivity [222,223]. P-gp is a plasma membrane protein that is encoded by the multidrug-resistant gene(s). An increasing number of studies show that ROS can regulate the expression of P-gp and can function as a negative regulator to downregulate P-gp expression [224,225]. A P-gp-based ATPase stimulation pathway has been supported by earlier studies and other recent evidence [223,226]. A non-P-gp-dependent pathway is also being gradually demonstrated in other studies. It is certain that ROS species have great potential and can eventually act as an agent to improve the condition of cancer treatments by improving drug sensitivity and solving the problem of drug resistance in a controlled way. As noted earlier, some important anticancer drugs show action by modulating the amount of ROS in cancer cells, and their action depends on the ROS. Moreover, the exact amount and concentration of ROS can enhance their action, which would be a blessing for those undergoing cancer treatments. The most valuable aspect to explore in the future can be the establishment of the amounts of ROS that are needed for enhancing drug sensitivity and playing a positive role in drug resistance. Discovering threshold limits to avoid the detrimental effects that can be caused by ROS during cancer treatment is another noble goal.

By following the ROS-inducing effect, some other important and potential methods can play significant roles in cancer treatment strategy. It has been reported that some monoclonal antibodies and tyrosine kinase inhibitors (TKIs) provide anticancer activity on patients via ROS-mediated mechanisms of action, which can also be related to their efficacy [227]. Again, there is another very promising therapy called sonodynamic therapy (SDT), which can enhance the level of ROS in cancer cells and affect the cancer microenvironment, which in turn can stop the development of cancer [228]. ROS is also a very good modulator of tumor-associated macrophages, which in turn evoke strong antitumor immune action resulting in the suppression of tumors [229]. The other promising ROS-based therapies are listed in Table 2.

## 5. Role of Plasma in the Inhibition of Cancer and its Mechanism

ROS originating in plasma (directly from plasma or subsequently produced in media) initially come into contact with the cytoplasmic membrane. Shortly after a plasma treatment at a sufficiently high dosage, numerous cancer cells undergo a morphological change from a broadened shape to a contractive shape [231,232].

Plasma contains a collection of ROS, and these ROS can encourage oxidative stress and activate different signaling pathways in cells. The primary mechanism of a non-thermal plasma anticancer treatment is related to ROS production. In a recent paper, Watson reported that ROS can serve as a ‘positive energy for life’ due to their function in apoptosis, i.e., as an inner program that extremely stresses cells to induce death [233]. On the other hand, ROS are also well recognized for their capability to irretrievably harm major proteins and nucleic acid molecules (DNA and RNA). It was also pointed out that the great majority of all the agents that are utilized to destroy cancer cells easily (ionizing radiation, most chemotherapeutic specialists, and some focused therapies) work by either straightforwardly or not directly producing ROS that obstruct key steps in the cellular cycle. It has been identified that a major boost of the intracellular ROS levels can cause DNA damage and apoptosis within the focused cells [14,233]. Preliminary observations also indicated that cancer cells consumed ROS much faster than other normal cells which supported the selective model based on aquaporins [234]. Plasma tends to resist the development of cancer cells, but not the development of homologous normal cells by activating more apoptosis in cancer cells than in ordinary cells [235,236]. Considering these selective anticancer methods is one of the key challenges in this area. Such a specific impact may be mainly due to the broad discovery that a recognizable rise of ROS specifically occurs in cancer cells rather than normal cells during a similar plasma treatment [121,237,238]. After the plasma treatment, the calculated ROS intensity in cancer cells is superior to that in normal cells. Nonetheless, in some instances, plasma kills more cancer cells than similar normal cells [36,239]

It is known that mitochondria are the key organelles that create ROS and the most common target of ROS-prompted damage, as discovered in different pathological states. In mitochondria, different types of ROS (mostly superoxide) are produced in three electron-transport chain complexes (Succinate-Q reductase, nicotinamide adenine dinucleotide phosphate-Q (NADP-Q) oxidoreductase, and Q-cytochrome oxidoreductase). As a counteract procedure, superoxide may be removed by targeting manganese (Mn)-reliant superoxide dismutase (MnSOD) in the matrix of mitochondria [21]. Although it is very challenging to target MnSOD only in cancer cells, some research works found evidence that there is high variability in MnSOD gene expression in cancer cells compared to normal cells; also, targeting MnSOD can be therapeutically beneficial for cancer. It has also been found that the promoter region of human MnSOD consists of peroxisome proliferator response element (PPRE)-binding motifs. Activation of the peroxisome proliferator-activated receptor-γ (PPARγ) in invasive basal-like breast cancer cell can eventually result in a significant lowering of MnSOD mRNA and protein levels, and it can be done by PPARγ ligands. The repression of MnSOD levels in cancer cells can control the intracellular ROS level in cancer cells [240].

**Figure 4:**
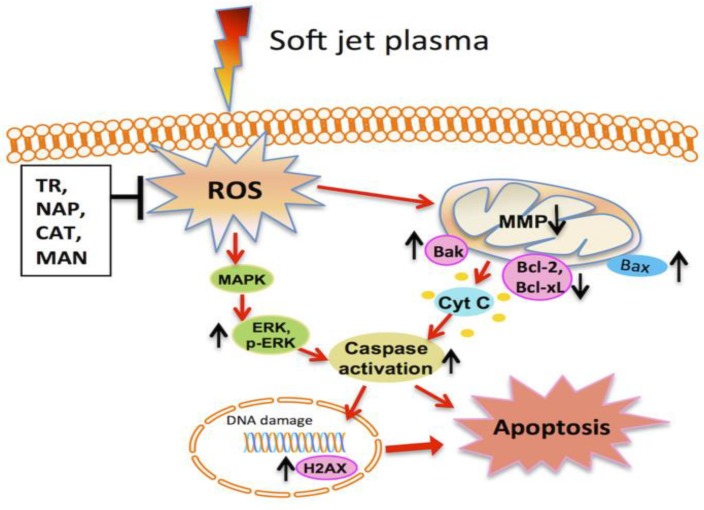
Molecular mechanism of soft-jet plasma-induced cancer cell apoptosis via the mitochondrial intrinsic pathway and extracellular signal-regulated kinase/mitogen-activated protein kinase (ERK/MAPK) activation [250].

Moreover, the phosphorylation of p53 is essential for triggering mitochondrion-based apoptosis pathways [241]. p53 activates the expression of pro-apoptotic components, including Bax, Puma, and Noxa [242]. These pro-apoptotic elements cause the discharge of cytochrome c and additional intermembrane mitochondrial proteins within the cytosol [243], where cytochrome c is linked to apoptotic protease actuating element1, later forming the apoptosome [244]. The apoptosome similarly actuates caspase-9 by means of cleavage [245,246]. The actuated caspase-9 advance enacts caspase-3/7 and eventually instigates the arrangement of apoptotic activities [245]. Among them, the cleavage of poly (ADP-ribose) polymerase (PARP) is a vital early molecular marker of apoptosis [245]. Apoptosis is the principal form of cancer cell death subsequent to a plasma treatment [246]. In plasma-treated cancer cells, the discharge of cytochrome c into the cytosol [232] and the appearance of caspase-3/7/9, Noxa [247], Bax, the PARP cleavage, mitochondrial transmembrane potential failure, as well as DNA destruction have been generally observed. In short, plasma-treated cancer cells not only follow distinctive DNA damage pathways [248], but they also maintain well-understood apoptosis pathways [249]. Figure 4 shows the possible molecular mechanisms of the apoptosis of soft jet plasma in cancer.

However, most apoptosis pathways observed in plasma-treated cancer cells are dependent on the mitochondrial mechanism activated through DNA injury and/or mitochondrial damage. DNA damage has generally been observed as a premature stage event during plasma treatment [234]. Double stranded break is the main damage type [22,251]. A vital marker of DSB is the particular phosphorylation of serine 139 on the H_2_AX histone (γ-H_2_AX), which is normally determined immediately after the plasma treatment [251]. Although apoptosis is the main and the most prominent pathway derived by plasma-based treatment, in some cancer cell lines, plasma treatment is reported to follow autophagy; both processes (autophagy and apoptosis) may have occurred simultaneously [252,253,254]. The enhanced oxidative stress has the ability to initiate autophagy. The redox signaling caused by the presence of ROS in cells can play a pivotal role in switching on autophagy. Again, we already discussed that DNA damage can be caused by plasma application, which can in turn cause DNA damage-induced autophagy, which can contribute as both a cell death and tumor-suppressor method [255]. In some cancer cell lines, it has been evident that plasma can cause the necrosis of cancer cells by high levels of DNA damage [256], but this is not as significant as the apoptosis mechanism.

In an endeavor to reveal in more detail the cascade of molecular activities that accompany a plasma treatment, human breast cancer cells were examined, and dose-based apoptosis appeared as a result of the plasma treatment [24]. Plasma effects including ROS production and the actuation of H_2_AX may also arise via an isolated treatment of culture media without cells and with a consequent switch to a condition with cells. The amount of DNA damage identified by the phosphorylated histone variant H_2_AX, which is recruited to DNA damage foci, was neither notably affected by the elimination of charged particles nor mediated by the UV content. Utilizing dilution experiments, researchers hypothesized that the cellular effects are interceded by way of the peroxidation of amino acids within the cell culture medium. Later, the authors verified that DNA damage is initiated by means of intracellular reactive species. The phosphorylation of H_2_AX appears to be especially interceded by the ataxia–telangiectasia associated protein (ATR) and not by the ataxia–telangiectasia mutated (ATM) form, which is mostly involved in the reaction between IR and H_2_O_2_. Moreover, ROS hinders the effects of plasma on in human liver cancer cells, where a noteworthy boost of lipid peroxidation was recognized. In addition, it has been discovered that intracellular ROS may also result in mitochondrial disorder [35].

Concentrating on cancer cells via ROS-mediated mechanisms has become an appealing method for the successful treatment of cancer while exploiting the abnormal redox characteristics of cancer cells [14]. While the levels of ROS in cancer cells are close to the limit at which cell death occurs and the sources of ROS formation in most cancer cells are dissimilar from those in normal cells [257], ROS have been investigated as anticancer remedial drugs. The opportunity for ROS upregulation through inhibitors of antioxidant enzymes or by means of ROS inducers has arisen, thus promoting oxidative stress and especially facilitating cancer cell death as anticancer healing agents [258]. It is important that plasma effects are clearer in several tumor types as compared to conventional chemotherapy [232]. Therefore, the collection of ROS directly initiated by plasma or through different mechanisms may present a novel basis on which a tumor remedy using plasma can be devised. We summarized some important studies on the effect of plasma toward cancer treatment in Table 3.

## 6. Future Perspective

At present, the most lethal and dangerous family of disease is cancer. The common day-to-day treatment of cancer is becoming more challenging due to the emergence of the harmful side effects [271] of cancer treatment strategies and the increasing resistance of therapeutics [272]. Hence, finding new methods for cancer treatment is an emerging topic that has attracted a vast number of scientists. In this review paper, we discussed how reactive species play a key role in cancer pathology and showed how these species have been targeted in cancer treatments. A wide range of anticancer medications can produce ROS, and they can be used to treat cancer by a number of different mechanisms. Most of the anticancer drugs act on cancer cells by producing ROS, and research should be performed to reduce the usual side effects of these cancer drugs. As the complications related to side effects are increasing, a prodrug specific to the cancer cells can be designed that can be initiated by an enhanced level of ROS present in cancer cells, and it can minimize the risk of unwanted side effects [273]. Similarly, the production of ROS can be targeted to develop a combinatorial treatment method of nanoparticles with anticancer drugs, which can provide a nanoparticle-based redox-directed combinational anticancer therapy to treat cancer [274]. Besides the conventional treatment methods, the alternative medicines are gaining popularity because of their lower possibility of causing treatment-related complications. However, some of the dietary active compounds have the ability to produce ROS, induce oxidative stress, and consequently cause cancer cell death. The application of alternative medicines to enhance ROS in cancer cells can be a very promising therapeutic strategy, but in order for this to take place, the foremost need is to enhance the bioavailability of the dietary compounds. Poor bioavailability is the main drawback of using dietary compounds. By improving all the pharmacokinetics parameters of these dietary compounds, it would be possible to develop them into a dosage form that can boost the ROS level effectively to treat cancer [275]. Photodynamic therapy is also a useful approach that can produce ROS. According to recent research works, improvements in nanotechnology and nanomedicine made it possible to develop ROS-generating systems by both photodynamic and non-photodynamic procedures, which create a possibility for photodynamic therapy to be applied as an anti-tumor agent [276].

Plasma is also a great source of ROS, and it can modulate a number of pathways in biological systems. Significant numbers of studies have already been performed to determine the efficacy and possible mechanisms of plasma in treating cancer and discover the roles of plasma in different types of cancer. It is now recognized that plasma can destroy cancer cells with selective killing effects toward cancer cells [35,38]. Accordingly, plasma technology can represent a ray of hope in the present situation of cancer treatments.

In different research works, different types of plasma devices have been used to treat cancer cells derived from different tumor types. Thus, it may be that plasma with different characteristics will react by different pathways in different types of cancer. The different pathways that are involved in this process are not fully understood. Due to the identical reactions of tumor cell types, it appears as if the same mechanisms are engaged in different tumor types. Therefore, in order to understand the anticancer mechanism of plasma, it is necessary to determine the molecular mechanisms of plasma acting on cancer cells.

In the present scenario, future works should be designed to find the most effective carriers to administer plasma as a therapy for patients. Several types of plasma instruments have been used for experiments on cell lines and on animals. It is now necessary to design the most effective types for treating patients, and new research works should focus on this. Again, in some studies, researchers found that plasma-treated media, solutions, and water show beneficial and cancer cell-killing effects. For these cases, we should find a suitable carrier or method by which to maintain the efficacy of the plasma-treated solutions.

As a treatment method or as a therapeutic strategy, we cannot ignore the possibilities of harmful side effects or the risk of toxicity. For this reason, it is now necessary to focus on the toxicological possibilities of plasma treatments on biological systems. Although plasma is known to be a non-toxic and non-harmful method, it can have certain long-term harmful or toxicological effects on humans, and these possibilities must be explored in more depth. If plasma can cause toxic outcomes in animal models after long-term treatment, then effective and safe dosage levels during plasma treatments should be established. This is why future research works should focus on discovering the pharmacokinetic parameters of plasma treatment technologies for their safe administration.

## Figures and Tables

**Table 1:** List of anticancer agents applied to different cancer treatment and their mechanism of action by increasing ROS production.

Published Year	Anticancer Agent	Types of Cancer	Mechanism
1999	Doxorubicin	Lung cancer [152]	BRAF inhibition by ROS [152,153]
2018	Actinomycin D or Decitabine	Skin cancer [154]	Production of reactive species [155]
2018	Vinorelbine	Lung cancer [156]	ROS induced mechanism [137]
2014	Vinblastine	Lung cancer and breast cancer [157]	Apoptosis induced by ROS [136]
2009	Camptothecin	Cervical and uterus cancer [158]	Cell death induced by ROS
2006 2014	Paclitaxel	Lung cancer [159] Breast cancer [160]	ROS-dependent activation of apoptotic cell death [161]
2012	Taxol	Blood cancer [162]	Apoptosis by generation of ROS [162]
2017	Epirubicin	Breast cancer [163]	Programmed death of cell by ROS [163]
2012	Resveratrol	Colon cancer [164]	ROS production [164]
2015	Colchicine	Colon cancer [165]	Increase ROS production in a dose dependent manner [165]

ROS: reactive oxygen species; BRAF: **serine/threonine-specific protein kinase**.

**Table 2:** List of other treatment methods used in cancer treatment via reactive oxygen species-based mechanisms.

Treatment Methods	Mechanism	Reference
Sonodynamic therapy (SDT)	Alter cancer microenvironment by enhancing ROS level	[228]
Tyrosin kinase inhibitor (TKI)	ROS inducing effect	[227]
Monoclonal antibody	ROS mediated apotosis	[227]
Anti-tumor immune action	By targeting tumor-associated macrophage by ROS	[229]
Nanomedicine combination with anticancer drugs	ROS-inducing effect	[230]

**Table 3:** List of plasma instruments and methods used in different types of cancer with their mechanisms.

Published Year	Plasma Equipment	Types of Cancer	Mechanism	Reference
2017	Plasma jet	Pancreatic cancer	Hydrogen peroxide	[259]
2017	DBD plasma device	Cervical cancer	Hydrogen peroxide	[260]
2014	Plasma jet	Head and neck cancer	DNA damage by ROS	[261]
2016	Plasma generated in DI water	Gastric cancer	ROS-induced apoptosis	[262]
2017	Air plasma by high voltage electrode	Triple negative breast cancer	Hydrogen peroxide-induced apoptosis	[256]
2016	Microplasma jet produced liquid plasma	Triple negative breast cancer	ROS and RNS-induced apoptosis	[263]
2017	DBD plasma device	Lung cancer	Apoptosis induced by ROS and RNS	[264]
2015	Water vapor with plasma jet	Breast cancer	Hydrogen peroxide-induced apoptosis	[265]
2017	DBD plasma	Colon cancer	Apoptosis and DNA damage by ROS	[266]
2013	Jet plasma	Brain cancer	Plasma caused cell death	[267]
2016	DBD plasma	Brain cancer	ROS-induced apoptosis	[268]
2012	DBD plasma	Brain and colorectal cancer	Apoptosis and DNA damage by ROS	[22]
2014	DBD plasma	Thyroid cancer, Oral cancer	ROS-induced DNA damage and apotosis	[238]
2013	Plasma-treated media	Blood cancer	ROS-induced apoptosis	[269]
2014	DBD plasma	Blood cancer	ROS-initiated apoptosis-related gene expression	[270]

DBD: dielectric barrier discharge; DI: deionized; ROS: reactive oxygen species; RNS: reactive nitrogen species.

## References

[1] Sauer H., Wartenberg M., Hescheler J. (2001). Reactive oxygen species as intracellular messengers during cell growth and differentiation. Cell. Physiol. Biochem..

[2] Halliwell B. (1991). Reactive oxygen species in living systems: Source, biochemistry, and role in human disease. Am. J. Med..

[3] Waris G., Ahsan H. (2006). Reactive oxygen species: Role in the development of cancer and various chronic conditions. J. Carcinog..

[4] Tong L., Chuang C.-C., Wu S., Zuo L. (2015). Reactive oxygen species in redox cancer therapy. Cancer Lett..

[5] Silva F., Marques A., Chaveiro A. (2010). Reactive oxygen species: A double-edged sword in reproduction. Open Vet. Sci. J..

[6] Pan J.-S., Hong M.-Z., Ren J.-L. (2009). Reactive oxygen species: A double-edged sword in oncogenesis. World J. Gastroenterol..

[7] Schumacker T. (2006). Reactive oxygen species in cancer cells: Live by the sword, die by the sword. Cancer Cell.

[8] Azad M.B., Chen Y., Gibson S.B. (2009). Regulation of autophagy by reactive oxygen species (ROS): Implications for cancer progression and treatment. Antioxid. Redox Signal..

[9] Raj L., Ide T., Gurkar A.U., Foley M., Schenone M., Li X., Tolliday N.J., Golub T.R., Carr S.A., Shamji A.F. (2011). Selective killing of cancer cells by a small molecule targeting the stress response to ROS. Nature.

[10] Circu M.L., Aw T.Y. (2010). Reactive oxygen species, cellular redox systems, and apoptosis. Free Radic. Biol. Med..

[11] Akhtar M.J., Ahamed M., Kumar S., Khan M.M., Ahmad J., Alrokayan S.A. (2012). Zinc oxide nanoparticles selectively induce apoptosis in human cancer cells through reactive oxygen species. Int. J. Nanomed..

[12] Dawson M.A., Kouzarides T. (2012). Cancer epigenetics: From mechanism to therapy. Cell.

[13] Hanahan D., Weinberg R.A. (2000). The hallmarks of cancer. Cell.

[14] Trachootham D., Alexandre J., Huang P. (2009). Targeting cancer cells by ROS-mediated mechanisms: A radical therapeutic approach?. Nat. Rev. Drug Discov..

[15] Leach J.K., Van Tuyle G., Lin P.-S., Schmidt-Ullrich R., Mikkelsen R.B. (2001). Ionizing radiation-induced, mitochondria-dependent generation of reactive oxygen/nitrogen. Cancer Res..

[16] Prise K.M., O’sullivan J.M. (2009). Radiation-induced bystander signalling in cancer therapy. Nat. Rev. Cancer.

[17] Mitra S., Dash R. (2018). Natural Products for the Management and Prevention of Breast Cancer. Evid.-Based Complement. Altern. Med..

[18] Lu X., Laroussi M., Puech V. (2012). On atmospheric-pressure non-equilibrium plasma jets and plasma bullets. Plasma Sources Sci. Technol..

[19] Kuchenbecker M., Bibinov N., Kaemlimg A., Wandke D., Awakowicz P., Viöl W. (2009). Characterization of DBD plasma source for biomedical applications. J. Phys. D Appl. Phys..

[20] Weltmann K., Von Woedtke T. (2016). Fusion, Plasma medicine—Current state of research and medical application. Plasma Phys. Control. Fusion.

[21] Ratovitski E.A., Cheng X., Yan D., Sherman J.H., Canady J., Trink B., Keidar M. (2014). Anti-cancer therapies of 21st century: Novel approach to treat human cancers using cold atmospheric plasma. Plasma Process. Polym..

[22] Vandamme M., Robert E., Lerondel S., Sarron V., Ries D., Dozias S., Sobilo J., Gosset D., Kieda C., Legrain B. (2012). ROS implication in a new antitumor strategy based on non-thermal plasma. Int. J. Cancer.

[23] Pai K., Timmons C., Roehm K.D., Ngo A., Narayanan S.S., Ramachandran A., Jacob J.D., Ma L.M., Madihally S.V. (2018). Investigation of the Roles of Plasma Species Generated by Surface Dielectric Barrier Discharge. Sci. Rep..

[24] Kalghatgi S., Kelly C.M., Cerchar E., Torabi B., Alekseev O., Fridman A., Friedman G., Azizkhan-Clifford J. (2011). Effects of non-thermal plasma on mammalian cells. PLoS ONE.

[25] Kim C.-H., Bahn J.H., Lee S.-H., Kim G.-Y., Jun S.-I., Lee K., Baek S.J. (2010). Induction of cell growth arrest by atmospheric non-thermal plasma in colorectal cancer cells. J. Biotechnol..

[26] Szatrowski T.P., Nathan C.F. (1991). Production of large amounts of hydrogen peroxide by human tumor cells. Cancer Res..

[27] Zhou Y., Hileman E.O., Plunkett W., Keating M.J., Huang P. (2003). Free radical stress in chronic lymphocytic leukemia cells and its role in cellular sensitivity to ROS-generating anticancer agents. Blood.

[28] Heinlin J., Isbary G., Stolz W., Morfill G., Landthaler M., Shimizu T., Steffes B., Nosenko T., Zimmermann J., Karrer S. (2011). Plasma applications in medicine with a special focus on dermatology. J. Eur. Acad. Dermatol. Venereol..

[29] Fridman G., Friedman G., Gutsol A., Shekhter A.B., Vasilets V.N., Fridman A. (2008). Applied plasma medicine. Plasma Process. Polym..

[30] Kong M.G., Kroesen G., Morfill G., Nosenko T., Shimizu T., Van Dijk J., Zimmermann J. (2009). Plasma medicine: An introductory review. New J. Phys..

[31] Koban I., Holtfreter B., Hübner N.O., Matthes R., Sietmann R., Kindel E., Weltmann K.D., Welk A., Kramer A., Kocher T. (2011). Antimicrobial efficacy of non-thermal plasma in comparison to chlorhexidine against dental biofilms on titanium discs in vitro–proof of principle experiment. J. Clin. Periodontol..

[32] Laroussi M. (2005). Low temperature plasma-based sterilization: Overview and state-of-the-art. Plasma Process. Polym..

[33] Schmidt A., Wende K., Bekeschus S., Bundscherer L., Barton A., Ottmüller K., Weltmann K.-D., Masur K. (2013). Non-thermal plasma treatment is associated with changes in transcriptome of human epithelial skin cells. Free Radic. Res..

[34] Wang M., Holmes B., Cheng X., Zhu W., Keidar M., Zhang L.G. (2013). Cold atmospheric plasma for selectively ablating metastatic breast cancer cells. PLoS ONE.

[35] Iseki S., Nakamura K., Hayashi M., Tanaka H., Kondo H., Kajiyama H., Kano H., Kikkawa F., Hori M. (2012). Selective killing of ovarian cancer cells through induction of apoptosis by nonequilibrium atmospheric pressure plasma. Appl. Phys. Lett..

[36] Hirst A., Simms M., Mann V., Maitland N., O’connell D., Frame F. (2015). Low-temperature plasma treatment induces DNA damage leading to necrotic cell death in primary prostate epithelial cells. Br. J. Cancer.

[37] Panngom K., Baik K., Nam M., Han J., Rhim H., Choi E. (2013). Preferential killing of human lung cancer cell lines with mitochondrial dysfunction by nonthermal dielectric barrier discharge plasma. Cell Death Dis..

[38] Tanaka H., Mizuno M., Ishikawa K., Nakamura K., Kajiyama H., Kano H., Kikkawa F., Hori M. (2011). Plasma-activated medium selectively kills glioblastoma brain tumor cells by down-regulating a survival signaling molecule, AKT kinase. Plasma Med..

[39] Fridman G., Shereshevsky A., Jost M.M., Brooks A.D., Fridman A., Gutsol A., Vasilets V., Friedman G. (2007). Floating electrode dielectric barrier discharge plasma in air promoting apoptotic behavior in melanoma skin cancer cell lines. Plasma Chem. Plasma Process..

[40] Kim G., Kim G., Park S., Jeon S., Seo H., Iza F., Lee J.K. (2008). Air plasma coupled with antibody-conjugated nanoparticles: A new weapon against cancer. J. Phys. D Appl. Phys..

[41] Kehrer J.P., Klotz L.O. (2015). Free radicals and related reactive species as mediators of tissue injury and disease: Implications for Health. Crit. Rev. Toxicol..

[42] Agnez-Lima L.F., Melo J.T., Silva A.E., Oliveira A.H.S., Timoteo A.R.S., Lima-Bessa K.M., Martinez G.R., Medeiros M.H., Di Mascio P., Galhardo R.S. (2012). DNA damage by singlet oxygen and cellular protective mechanisms. Mutat. Res./Rev. Mutat. Res..

[43] Nosaka Y., Nosaka A.Y. (2017). Generation and detection of reactive oxygen species in photocatalysis. Chem. Rev..

[44] Hayyan M., Hashim M.A., AlNashef I.M. (2016). Superoxide ion: Generation and chemical implications. Chem. Rev..

[45] Koppenol W.H. (2001). The Haber-Weiss cycle—70 years later. Redox Rep..

[46] Gligorovski S., Strekowski R., Barbati S., Vione D. (2015). Environmental implications of hydroxyl radicals (• OH). Chem. Rev..

[47] Luis A., Sandalio L.M., Palma J., Bueno P., Corpas F.J. (1992). Metabolism of oxygen radicals in peroxisomes and cellular implications. Free Radic. Biol. Med..

[48] Finkel T. (2011). Signal transduction by reactive oxygen species. J. Cell Biol..

[49] Quan L.J., Zhang B., Shi W.W., Li H.Y. (2008). Hydrogen peroxide in plants: A versatile molecule of the reactive oxygen species network. J. Integr. Plant Biol..

[50] Schieber M., Chandel N.S. (2014). ROS function in redox signaling and oxidative stress. Curr. Biol..

[51] Apel K., Hirt H. (2004). Reactive oxygen species: Metabolism, oxidative stress, and signal transduction. Annu. Rev. Plant Biol..

[52] Crichton R., Crichton R.R., Boelaert J.R. (2001). Inorganic Biochemistry of Iron Metabolism: From Molecular Mechanisms to Clinical Consequences.

[53] Cadenas E. (1989). Biochemistry of oxygen toxicity. Annu. Rev. Biochem..

[54] Halliwell B. (1989). Protection against oxidants in biological systems. The superoxide theory of oxygen toxicity. Free Radicals in Biology and Medicine.

[55] Klotz L.-O. (2002). Oxidant-induced signaling: Effects of peroxynitrite and singlet oxygen. Biol. Chem..

[56] Feig D.I., Reid T.M., Loeb L.A. (1994). Reactive oxygen species in tumorigenesis. Cancer Res..

[57] Klaunig J.E., Xu Y., Isenberg J.S., Bachowski S., Kolaja K.L., Jiang J., Stevenson D.E., Walborg E.F. (1998). The role of oxidative stress in chemical carcinogenesis. Environ. Health Perspect..

[58] Kim Y.-W., West X.Z., Byzova T.V. (2013). Inflammation and oxidative stress in angiogenesis and vascular disease. J. Mol. Med..

[59] Shelton P., Jaiswal A.K. (2013). The transcription factor NF-E2-related factor 2 (Nrf2): A protooncogene?. FASEB J..

[60] St-Pierre J., Buckingham J.A., Roebuck S.J., Brand M.D. (2002). Topology of superoxide production from different sites in the mitochondrial electron transport chain. J. Biol. Chem..

[61] Ray D., Huang B.-W., Tsuji Y. (2012). Reactive oxygen species (ROS) homeostasis and redox regulation in cellular signaling. Cell. Signal..

[62] Sena L.A., Chandel N.S. (2012). Physiological roles of mitochondrial reactive oxygen species. Mol. Cell.

[63] Ott M., Gogvadze V., Orrenius S., Zhivotovsky B. (2007). Mitochondria, oxidative stress and cell death. Apoptosis.

[64] Naudi A., Jove M., Ayala V., Cassanye A., Serrano J., Gonzalo H., Boada J., Prat J., Portero-Otin M., Pamplona R. (2012). Cellular dysfunction in diabetes as maladaptive response to mitochondrial oxidative stress. Exp. Diabetes Res..

[65] Chance B., Sies H., Boveris A. (1979). Hydroperoxide metabolism in mammalian organs. Physiol. Rev..

[66] Okado-Matsumoto A., Fridovich I. (2001). Subcellular distribution of superoxide dismutases (SOD) in rat liver Cu, Zn-SOD in mitochondria. J. Biol. Chem..

[67] Sturtz L.A., Diekert K., Jensen L.T., Lill R., Culotta V.C. (2001). A fraction of yeast cu, zn-superoxide dismutase and its metallochaperone, ccs, localize to the intermembrane space of mitochondria a physiological role for sod1 in guarding against mitochondrial oxidative damage. J. Biol. Chem..

[68] Weisiger R.A., Fridovich I. (1973). Superoxide dismutase organelle specificity. J. Biol. Chem..

[69] Murphy M.P. (2009). How mitochondria produce reactive oxygen species. Biochem. J..

[70] Brand M.D. (2010). The sites and topology of mitochondrial superoxide production. Exp. Gerontol..

[71] Zangar R.C., Davydov D.R., Verma S. (2004). Mechanisms that regulate production of reactive oxygen species by cytochrome P450. Toxicol. Appl. Pharmacol..

[72] Cao S.S., Kaufman R.J. (2014). Endoplasmic reticulum stress and oxidative stress in cell fate decision and human disease. Antioxid. Redox Signal..

[73] Creighton T.E., Hillson D.A., Freedman R.B. (1980). Catalysis by protein-disulphide isomerase of the unfolding and refolding of proteins with disulphide bonds. J. Mol. Biol..

[74] Tu B.P., Weissman J.S. (2004). Oxidative protein folding in eukaryotes: Mechanisms and consequences. J. Cell Biol..

[75] Kemp M., Go Y.-M., Jones D.P. (2008). Nonequilibrium thermodynamics of thiol/disulfide redox systems: A perspective on redox systems biology. Free Radic. Biol. Med..

[76] Hwang C., Sinskey A.J., Lodish H.F. (1992). Oxidized redox state of glutathione in the endoplasmic reticulum. Science.

[77] Montezano A.C., Touyz R.M. (2012). Reactive oxygen species and endothelial function—Role of nitric oxide synthase uncoupling and Nox family nicotinamide adenine dinucleotide phosphate oxidases. Basic Clin. Pharmacol. Toxicol..

[78] Barnham K.J., Masters C.L., Bush A.I. (2004). Neurodegenerative diseases and oxidative stress. Nat. Rev. Drug Discov..

[79] Forman H.J., Torres M. (2002). Reactive oxygen species and cell signaling: Respiratory burst in macrophage signaling. Am. J. Respir. Crit. Care Med..

[80] Schrader M., Fahimi H.D. (2006). Peroxisomes and oxidative stress. Biochim. Biophys. Acta (BBA)-Mol. Cell Res..

[81] Abdal Dayem A., Hossain M.K., Lee S.B., Kim K., Saha S.K., Yang G.-M., Choi H.Y., Cho S.-G. (2017). The role of reactive oxygen species (ROS) in the biological activities of metallic nanoparticles. Int. J. Mol. Sci..

[82] Hrycay E.G., Bandiera S.M. (2015). Involvement of cytochrome P450 in reactive oxygen species formation and cancer. Adv. Pharmacol..

[83] Halliwell B. (2006). Oxidative stress and neurodegeneration: Where are we now?. J. Neurochem..

[84] Hardwick J.P. (2015). Cytochrome P450 Function and Pharmacological Roles in Inflammation and Cancer.

[85] Hrycay E.G., Bandiera S.M. (2012). The monooxygenase, peroxidase, and peroxygenase properties of cytochrome P450. Arch. Biochem. Biophys..

[86] Lewis D., Lake B. (2002). Species differences in coumarin metabolism: A molecular modelling evaluation of CYP2A interactions. Xenobiotica.

[87] Kappus H. (1993). Metabolic reactions: Role of cytochrome P-450 in the formation of reactive oxygen species. Cytochrome P450.

[88] Bagchi D., Bagchi M., Hassoun E., Stohs S. (1995). In vitro and in vivo generation of reactive oxygen species, DNA damage and lactate dehydrogenase leakage by selected pesticides. Toxicology.

[89] Wilhelm J., Sojkova J., Herget J. (1995). Production of hydrogen peroxide by alveolar macrophages: Effect of barbiturates. Physiol. Res..

[90] Datta R., Yoshinaga K., Kaneki M., Pandey P., Kufe D. (2000). Phorbol ester-induced generation of reactive oxygen species is protein kinase Cβ-dependent and required for SAPK activation. J. Biol. Chem..

[91] Ice J.M., Diwan B.A., Hu H., Ward J.M., Nims R.W., Lubet R.A. (1994). Enhancement of hepatocarcinogenesis and induction of specific cytochrome P450-dependent monooxygenase activities by the barbiturates allobarbital, aprobarbital, pentobarbital, secobarbital and 5-phenyl-and 5-ethylbarbituric acids. Carcinogenesis.

[92] Klaunig J.E., Kamendulis L.M. (2004). The role of oxidative stress in carcinogenesis. Annu. Rev. Pharmacol. Toxicol..

[93] Siesky A.M., Kamendulis L.M., Klaunig J.E. (2002). Hepatic effects of 2-butoxyethanol in rodents. Toxicol. Sci..

[94] Kitazawa M., Anantharam V., Kanthasamy A.G. (2001). Dieldrin-induced oxidative stress and neurochemical changes contribute to apoptopic cell death in dopaminergic cells. Free Radic. Biol. Med..

[95] Kopf P.G., Walker M. (2010). 2, 3, 7, 8-tetrachlorodibenzo-p-dioxin increases reactive oxygen species production in human endothelial cells via induction of cytochrome P4501A1. Toxicol. Appl. Pharmacol..

[96] Betoulle S., Duchiron C., Deschaux P. (2000). Lindane increases in vitro respiratory burst activity and intracellular calcium levels in rainbow trout (Oncorhynchus mykiss) head kidney phagocytes. Aquat. Toxicol..

[97] Kinoshita A., Wanibuchi H., Imaoka S., Ogawa M., Masuda C., Morimura K., Funae Y., Fukushima S. (2002). Formation of 8-hydroxydeoxyguanosine and cell-cycle arrest in the rat liver via generation of oxidative stress by phenobarbital: Association with expression profiles of p21WAF1/Cip1, cyclin D1 and Ogg1. Carcinogenesis.

[98] Klaunig J.E., Xu Y., Bachowski S., Jiang J. (1997). Free-radical oxygen-induced changes in chemical carcinogenesis. Free Radical Toxicology.

[99] Formanowicz D., Radom M., Rybarczyk A., Formanowicz P. (2018). The role of Fenton reaction in ROS-induced toxicity underlying atherosclerosis–modeled and analyzed using a Petri net-based approach. Biosystems.

[100] Pignatello J.J., Oliveros E., MacKay A. (2006). Advanced oxidation processes for organic contaminant destruction based on the Fenton reaction and related chemistry. Crit. Rev. Environ. Sci. Technol..

[101] Sutton H.C., Winterbourn C.C. (1989). On the participation of higher oxidation states of iron and copper in Fenton reactions. Free Radic. Biol. Med..

[102] Kehrer J.P. (2000). The Haber–Weiss reaction and mechanisms of toxicity. Toxicology.

[103] Kanti Das T., Wati M.R., Fatima-Shad K. (2014). Oxidative stress gated by Fenton and Haber Weiss reactions and its association with Alzheimer’s disease. Arch. Neurosci..

[104] Riley P.A. (1994). Free radicals in biology: Oxidative stress and the effects of ionizing radiation. Int. J. Radiat. Biol..

[105] Adhami V.M., Afaq F., Ahmad N. (2003). Suppression of ultraviolet B exposure-mediated activation of NF-κB in normal human keratinocytes by resveratrol. Neoplasia.

[106] Heck D.E., Vetrano A.M., Mariano T.M., Laskin J.D. (2003). UVB light stimulates production of reactive oxygen species: Unexpected role for catalase. J. Biol. Chem..

[107] Baskar R., Dai J., Wenlong N., Yeo R., Yeoh K.-W. (2014). Biological response of cancer cells to radiation treatment. Front. Mol. Biosci..

[108] Azzam E.I., Jay-Gerin J.-P., Pain D. (2012). Ionizing radiation-induced metabolic oxidative stress and prolonged cell injury. Cancer Lett..

[109] Tateishi Y., Sasabe E., Ueta E., Yamamoto T. (2008). Ionizing irradiation induces apoptotic damage of salivary gland acinar cells via NADPH oxidase 1-dependent superoxide generation. Biochem. Biophys. Res. Commun..

[110] Chen Q., Chai Y., Mazumder S., Jiang C., Macklis R., Chisolm G., Almasan A. (2003). The late increase in intracellular free radical oxygen species during apoptosis is associated with cytochrome c release, caspase activation, and mitochondrial dysfunction. Cell Death Differ..

[111] Yamamori T., Yasui H., Yamazumi M., Wada Y., Nakamura Y., Nakamura H., Inanami O. (2012). Ionizing radiation induces mitochondrial reactive oxygen species production accompanied by upregulation of mitochondrial electron transport chain function and mitochondrial content under control of the cell cycle checkpoint. Free Radic. Biol. Med..

[112] Farivar S., Malekshahabi T., Shiari R. (2014). Biological effects of low level laser therapy. J. Lasers Med. Sci..

[113] Karu T. (1987). Photobiological fundamentals of low-power laser therapy. IEEE J. Quantum Electron..

[114] Eells J.T., Wong-Riley M.T., VerHoeve J., Henry M., Buchman E.V., Kane M.P., Gould L.J., Das R., Jett M., Hodgson B.D. (2004). Mitochondrial signal transduction in accelerated wound and retinal healing by near-infrared light therapy. Mitochondrion.

[115] Karu T., Afanas’eva N. (1995). Cytochrome c oxidase as a primary photoacceptor when laser irradiating cell culture by visible and near IR-range light. Doklady Akademii Nauk-Rossijskaya Akademiya Nauk.

[116] Conlan M.J., Rapley J.W., Cobb C.M. (1996). Biostimulation of wound healing by low-energy laser irradiation A review. J. Clin. Periodontol..

[117] Mester E., Mester A.F., Mester A. (1985). The biomedical effects of laser application. Lasers Surg. Med..

[118] Ortiz M.C.S., Carrinho P.M., Santos A., Gonçalves R.C., Parizotto N.A. (2001). Laser de baixa intensidade: Princípios e generalidades–Parte 1. Fisioter. Bras..

[119] Stadler I., Evans R., Kolb B., Naim J.O., Narayan V., Buehner N., Lanzafame R.J. (2000). In vitro effects of low-level laser irradiation at 660 nm on peripheral blood lymphocytes. Lasers Surg. Med..

[120] Silveira P., Silva L., Tuon T., Freitas T., Streck E., Pinho R. (2009). Effects of low-level laser therapy on epidermal oxidative response induced by wound healing. Braz. J. Phys. Ther..

[121] Ja Kim S., Min Joh H., Chung T. (2013). Production of intracellular reactive oxygen species and change of cell viability induced by atmospheric pressure plasma in normal and cancer cells. Appl. Phys. Lett..

[122] Sousa J.S., Tresp H., Dünnbier M., Iséni S., Hammer M.U., Winter J., Martin V., Puech V., Weltmann K.-D., Reuter S. Plasma-generated reactive oxygen species for biomedical applications. Proceedings of the 4th International Conference on Plasma Medicine.

[123] Klämpfl T.G., Isbary G., Shimizu T., Li Y.-F., Zimmermann J.L., Stolz W., Schlegel J., Morfill G.E., Schmidt H.-U. (2012). Cold atmospheric air plasma sterilization against spores and other microorganisms of clinical interest. Appl. Environ. Microbiol..

[124] Leclaire C., Lecoq E., Orial G., Clement F., Bousta F. Fungal decontamination by cold plasma: An innovating process for wood treatment. Proceedings of the COST Action IE0601/ESWM-International Conference.

[125] Keidar M., Walk R., Shashurin A., Srinivasan P., Sandler A., Dasgupta S., Ravi R., Guerrero-Preston R., Trink B. (2011). Cold plasma selectivity and the possibility of a paradigm shift in cancer therapy. Br. J. Cancer.

[126] Ahn H.J., Kim K.I., Hoan N.N., Kim C.H., Moon E., Choi K.S., Yang S.S., Lee J.-S. (2014). Targeting cancer cells with reactive oxygen and nitrogen species generated by atmospheric-pressure air plasma. PLoS ONE.

[127] Kumar N., Attri P., Yadav D.K., Choi J., Choi E.H., Uhm H.S. (2014). Induced apoptosis in melanocytes cancer cell and oxidation in biomolecules through deuterium oxide generated from atmospheric pressure non-thermal plasma jet. Sci. Rep..

[128] Yan D., Sherman J.H., Keidar M. (2017). Cold atmospheric plasma, a novel promising anti-cancer treatment modality. Oncotarget.

[129] Ahn H.J., Kim K.I., Kim G., Moon E., Yang S.S., Lee J.-S. (2011). Atmospheric-pressure plasma jet induces apoptosis involving mitochondria via generation of free radicals. PLoS ONE.

[130] Gorrini C., Harris I.S., Mak T.W. (2013). Modulation of oxidative stress as an anticancer strategy. Nat. Rev. Drug Discov..

[131] Wen J., You K.-R., Lee S.-Y., Song C.-H., Kim D.-G. (2002). Oxidative stress-mediated apoptosis the anticancer effect of the sesquiterpene lactone parthenolide. J. Biol. Chem..

[132] Lim J.-H., Lee Y.-M., Park S.R., DA HYE K., Lim B.O. (2014). Anticancer activity of hispidin via reactive oxygen species-mediated apoptosis in colon cancer cells. Anticancer Res..

[133] Pervaiz S., Clement M.-V. (2007). Superoxide anion: Oncogenic reactive oxygen species?. Int. J. Biochem. Cell Biol..

[134] McKeague A., Wilson D., Nelson J. (2003). Staurosporine-induced apoptosis and hydrogen peroxide-induced necrosis in two human breast cell lines. Br. J. Cancer.

[135] Simizu S., Takada M., Umezawa K., Imoto M. (1998). Requirement of caspase-3(-like) protease-mediated hydrogen peroxide production for apoptosis induced by various anticancer drugs. J. Biol. Chem..

[136] Yokoyama C., Sueyoshi Y., Ema M., Mori Y., Takaishi K., Hisatomi H. (2017). Induction of oxidative stress by anticancer drugs in the presence and absence of cells. Oncol. Lett..

[137] Yamada T., Egashira N., Imuta M., Yano T., Yamauchi Y., Watanabe H., Oishi R. (2010). Role of oxidative stress in vinorelbine-induced vascular endothelial cell injury. Free Radic. Biol. Med..

[138] Mercuro G., Cadeddu C., Piras A., Dessi M., Madeddu C., Deidda M., Serpe R., Massa E., Mantovani G. (2007). Early epirubicin-induced myocardial dysfunction revealed by serial tissue Doppler echocardiography: Correlation with inflammatory and oxidative stress markers. Oncologist.

[139] Lo Y.-L., Wang W. (2013). Formononetin potentiates epirubicin-induced apoptosis via ROS production in HeLa cells in vitro. Chem.-Biol. Interact..

[140] Gilliam L.A., Moylan J.S., Patterson E.W., Smith J.D., Wilson A.S., Rabbani Z., Reid M.B. (2011). Doxorubicin acts via mitochondrial ROS to stimulate catabolism in C2C12 myotubes. Am. J. Physiol.-Cell Physiol..

[141] Minotti G., Menna P., Salvatorelli E., Cairo G., Gianni L. (2004). Anthracyclines: Molecular advances and pharmacologic developments in antitumor activity and cardiotoxicity. Pharmacol. Rev..

[142] Mizutani H. (2007). Mechanism of DNA damage and apoptosis induced by anticancer drugs through generation of reactive oxygen species. Yakugaku zasshi J. Pharm. Soc. Jpn..

[143] Li L., Yue G., Pu J., Sun H., Fung K., Leung P., Han Q., Lau C., Leung P. (2014). Eriocalyxin B-induced apoptosis in pancreatic adenocarcinoma cells through thiol-containing antioxidant systems and downstream signalling pathways. Curr. Mol. Med..

[144] Noori S., Hassan Z.M., Farsam V. (2014). Artemisinin as a Chinese medicine, selectively induces apoptosis in pancreatic tumor cell line. Chin. J. Integr. Med..

[145] Dando I., Fiorini C., Dalla Pozza E., Padroni C., Costanzo C., Palmieri M., Donadelli M. (2013). UCP2 inhibition triggers ROS-dependent nuclear translocation of GAPDH and autophagic cell death in pancreatic adenocarcinoma cells. Biochim. Biophys. Acta (BBA)-Mol. Cell Res..

[146] Chen S.-H., Li D.-L., Yang F., Wu Z., Zhao Y.-Y., Jiang Y. (2014). Gemcitabine-induced pancreatic cancer cell death is associated with MST1/Cyclophilin D mitochondrial complexation. Biochimie.

[147] Zhao W., Li D., Liu Z., Zheng X., Wang J., Wang E. (2013). Spiclomazine induces apoptosis associated with the suppression of cell viability, migration and invasion in pancreatic carcinoma cells. PLoS ONE.

[148] Wang B., Wang X.-B., Chen L.-Y., Huang L., Dong R.-Z. (2013). Belinostat-induced apoptosis and growth inhibition in pancreatic cancer cells involve activation of TAK1-AMPK signaling axis. Biochem. Biophys. Res. Commun..

[149] Du J.-H., Zhang H.-D., Ma Z.-J., Ji K.-M. (2010). Artesunate induces oncosis-like cell death in vitro and has antitumor activity against pancreatic cancer xenografts in vivo. Cancer Chemother. Pharmacol..

[150] Khan M., Ding C., Rasul A., Yi F., Li T., Gao H., Gao R., Zhong L., Zhang K., Fang X. (2012). Isoalantolactone induces reactive oxygen species mediated apoptosis in pancreatic carcinoma PANC-1 cells. Int. J. Biol. Sci..

[151] Kong R., Jia G., Cheng Z.-X., Wang Y.-W., Mu M., Wang S.-J., Pan S.-H., Gao Y., Jiang H.-C., Dong D.-L. (2012). Dihydroartemisinin enhances Apo2L/TRAIL-mediated apoptosis in pancreatic cancer cells via ROS-mediated up-regulation of death receptor 5. PLoS ONE.

[152] Han J.A., Park S.C. (1999). Hydrogen peroxide mediates doxorubicin-induced transglutaminase 2 expression in PC-14 human lung cancer cell line. Exp. Mol. Med..

[153] Kim S.-Y., Kim S.-J., Kim B.-J., Rah S.-Y., Chung S.M., Im M.-J., Kim U.-H. (2006). Doxorubicin-induced reactive oxygen species generation and intracellular Ca2+ increase are reciprocally modulated in rat cardiomyocytes. Exp. Mol. Med..

[154] Yuan L., Mishra R., Patel H., Abdulsalam S., Greis K.D., Kadekaro A.L., Merino E.J., Garrett J.T. (2018). Utilization of Reactive Oxygen Species Targeted Therapy to Prolong the Efficacy of BRAF Inhibitors in Melanoma. J. Cancer.

[155] Brodská B., Holoubek A. (2011). Generation of reactive oxygen species during apoptosis induced by DNA-damaging agents and/or histone deacetylase inhibitors. Oxid. Med. Cell. Longev..

[156] Wu Y.-W., Lin C.-F., Lin Y.-S., Su W.-C., Chiu W.-H. (2018). Autophagy regulates vinorelbine sensitivity due to continued Keap1-mediated ROS generation in lung adenocarcinoma cells. Cell Death Discov..

[157] Samadi N., Ghanbari P., Mohseni M., Tabasinezhad M., Sharifi S., Nazemieh H., Rashidi M.R. (2014). Combination therapy increases the efficacy of docetaxel, vinblastine and tamoxifen in cancer cells. J. Cancer Res. Ther..

[158] Ha S.W., Kim Y.J., Kim W., Lee C.S. (2009). Antitumor Effects of Camptothecin Combined with Conventional Anticancer Drugs on the Cervical and Uterine Squamous Cell Carcinoma Cell Line SiHa. Korean J. Physiol. Pharmacol..

[159] Alexandre J., Batteux F., Nicco C., Chereau C., Laurent A., Guillevin L., Weill B., Goldwasser F. (2006). Accumulation of hydrogen peroxide is an early and crucial step for paclitaxel-induced cancer cell death both in vitro and in vivo. Int. J. Cancer.

[160] Pan Z., Avila A., Gollahon L. (2014). Paclitaxel Induces Apoptosis in Breast Cancer Cells through Different Calcium—Regulating Mechanisms Depending on External Calcium Conditions. Int. J. Mol. Sci..

[161] Kim H.S., Oh J.M., Jin D.H., Yang K.-H., Moon E.-Y. (2008). Paclitaxel induces vascular endothelial growth factor expression through reactive oxygen species production. Pharmacology.

[162] Meshkini A., Yazdanparast R. (2012). Involvement of oxidative stress in taxol-induced apoptosis in chronic myelogenous leukemia K562 cells. Exp. Toxicol. Pathol..

[163] Liu L., Mu L.-M., Yan Y., Wu J.-S., Hu Y.-J., Bu Y.-Z., Zhang J.-Y., Liu R., Li X.-Q., Lu W.-L. (2017). The use of functional epirubicin liposomes to induce programmed death in refractory breast cancer. Int. J. Nanomed..

[164] Miki H., Uehara N., Kimura A., Sasaki T., Yuri T., Yoshizawa K., Tsubura A. (2012). Resveratrol induces apoptosis via ROS-triggered autophagy in human colon cancer cells. Int. J. Oncol..

[165] Huang Z., Xu Y., Peng W. (2015). Colchicine induces apoptosis in HT-29 human colon cancer cells via the AKT and c-Jun N-terminal kinase signaling pathways. Mol. Med. Rep..

[166] Davies K.J.A. (1999). The Broad Spectrum of Responses to Oxidants in Proliferating Cells: A New Paradigm for Oxidative Stress. IUBMB Life.

[167] Matés J.M., Sánchez-Jiménez F.M. (2000). Role of reactive oxygen species in apoptosis: Implications for cancer therapy. Int. J. Biochem. Cell Biol..

[168] Kong Q., Beel J.A., Lillehei K.O. (2000). A threshold concept for cancer therapy. Med. Hypotheses.

[169] Amri F., Ghouili I., Amri M., Carrier A., Masmoudi-Kouki O. (2017). Neuroglobin protects astroglial cells from hydrogen peroxide-induced oxidative stress and apoptotic cell death. J. Neurochem..

[170] Weinberg S.E., Chandel N.S. (2015). Targeting mitochondria metabolism for cancer therapy. Nat. Chem. Biol..

[171] Cairns R.A., Harris I.S., Mak T.W. (2011). Regulation of cancer cell metabolism. Nat. Rev. Cancer.

[172] Cook J.A., Gius D., Wink D.A., Krishna M.C., Russo A., Mitchell J.B. (2004). Oxidative stress, redox, and the tumor microenvironment. Seminars in Radiation Oncology.

[173] Poljsak B., Šuput D., Milisav I. (2013). Achieving the balance between ROS and antioxidants: When to use the synthetic antioxidants. Oxid. Med. Cell. Longev..

[174] Schafer F.Q., Buettner G.R. (2001). Redox environment of the cell as viewed through the redox state of the glutathione disulfide/glutathione couple. Free Radic. Biol. Med..

[175] Holmgren A. (2000). Antioxidant function of thioredoxin and glutaredoxin systems. Antioxid. Redox Signal..

[176] Landriscina M., Maddalena F., Laudiero G., Esposito F. (2009). Adaptation to oxidative stress, chemoresistance, and cell survival. Antioxid. Redox Signal..

[177] Pelicano H., Carney D., Huang P. (2004). ROS stress in cancer cells and therapeutic implications. Drug Resist. Updat..

[178] Sastre J., Pallardó F.V., Viña J. (2000). Mitochondrial oxidative stress plays a key role in aging and apoptosis. IUBMB Life.

[179] Carmody R.J., Cotter T.G. (2001). Signalling apoptosis: A radical approach. Redox Rep..

[180] Hensley K., Robinson K.A., Gabbita S.P., Salsman S., Floyd R.A. (2000). Reactive oxygen species, cell signaling, and cell injury. Free Radic. Biol. Med..

[181] Levine R.L., Stadtman E.R. (2001). Oxidative modification of proteins during aging. Exp. Gerontol..

[182] Mallis R.J., Thomas J.A. (2001). Oxidative modification of H-ras: S-thiolation and S-nitrosylation of reactive cysteines. Biochem. J..

[183] Zhang J., Wang X., Vikash V., Ye Q., Wu D., Liu Y., Dong W. (2016). ROS and ROS-mediated cellular signaling. Oxid. Med. Cell. Longev..

[184] Ozben T. (2007). Oxidative stress and apoptosis: Impact on cancer therapy. J. Pharm. Sci..

[185] Saitoh M., Nishitoh H., Fujii M., Takeda K., Tobiume K., Sawada Y., Kawabata M., Miyazono K., Ichijo H. (1998). Mammalian thioredoxin is a direct inhibitor of apoptosis signal-regulating kinase (ASK) 1. EMBO J..

[186] Takeda K., Matsuzawa A., Nishitoh H., Ichijo H. (2003). Roles of MAPKKK ASK1 in stress-induced cell death. Cell Struct. Funct..

[187] Brunet A., Bonni A., Zigmond M.J., Lin M.Z., Juo P., Hu L.S., Anderson M.J., Arden K.C., Blenis J., Greenberg M.E. (1999). Akt promotes cell survival by phosphorylating and inhibiting a Forkhead transcription factor. Cell.

[188] You H., Yamamoto K., Mak T.W. (2006). Regulation of transactivation-independent proapoptotic activity of p53 by FOXO3a. Proc. Natl. Acad. Sci. USA.

[189] Morgan M.J., Kim Y.-S., Liu Z.-G. (2008). TNFα and reactive oxygen species in necrotic cell death. Cell Res..

[190] Schulze-Osthoff K., Beyaert R., Vandevoorde V., Haegeman G., Fiers W. (1993). Depletion of the mitochondrial electron transport abrogates the cytotoxic and gene-inductive effects of TNF. EMBO J..

[191] Xu Y.C., Wu R.F., Gu Y., Yang Y.-S., Yang M.-C., Nwariaku F.E., Terada L.S. (2002). Involvement of TRAF4 in oxidative activation of c-Jun N-terminal kinase. J. Biol. Chem..

[192] Wong G., Goeddel D.V. (1988). Induction of manganous superoxide dismutase by tumor necrosis factor: Possible protective mechanism. Science.

[193] Halliwell B., Gutteridge J. (1999). Free radicals, other reactive species and disease. Free Radic. Biol. Med..

[194] Zdolsek J.M., Svensson I. (1993). Effect of reactive oxygen species on lysosomal membrane integrity. Virchows Archiv. B.

[195] Kroemer G., Reed J.C. (2000). Mitochondrial control of cell death. Nat. Med..

[196] Brown G.C., Borutaite V. (2002). Nitric oxide inhibition of mitochondrial respiration and its role in cell death. Free Radic. Biol. Med..

[197] Sade H., Krishna S., Sarin A. (2004). The anti-apoptotic effect of Notch-1 requires p56lck-dependent, Akt/PKB-mediated signaling in T cells. J. Biol. Chem..

[198] Kroemer G., Dallaporta B., Resche-Rigon M. (1998). The mitochondrial death/life regulator in apoptosis and necrosis. Annu. Rev. Physiol..

[199] Honeychurch J., Alduaij W., Azizyan M., Cheadle E.J., Pelicano H., Ivanov A., Huang P., Cragg M.S., Illidge T.M. (2012). Antibody-induced non-apoptotic cell death in human lymphoma and leukemia cells is mediated through a novel reactive oxygen species dependent pathway. Blood.

[200] Yang Y., Zhang Y., Wang L., Lee S. (2017). Levistolide a induces apoptosis via ROS-mediated ER stress pathway in colon cancer cells. Cell. Physiol. Biochem..

[201] Díaz-Laviada I., Rodríguez-Henche N. (2014). The potential antitumor effects of capsaicin. Capsaicin as a Therapeutic Molecule.

[202] Medan D., Wang L., Toledo D., Lu B., Stehlik C., Jiang B.H., Shi X., Rojanasakul Y. (2005). Regulation of Fas (CD95)-induced apoptotic and necrotic cell death by reactive oxygen species in macrophages. J. Cell. Physiol..

[203] Uchikura K., Wada T., Hoshino S., Nagakawa Y., Aiko T., Bulkley G.B., Klein A.S., Sun Z. (2004). Lipopolysaccharides induced increases in Fas ligand expression by Kupffer cells via mechanisms dependent on reactive oxygen species. Am. J. Physiol.-Gastrointest. Liver Physiol..

[204] Hunter D.J., Kraft P., Jacobs K.B., Cox D.G., Yeager M., Hankinson S.E., Wacholder S., Wang Z., Welch R., Hutchinson A. (2007). A genome-wide association study identifies alleles in FGFR2 associated with risk of sporadic postmenopausal breast cancer. Nat. Genet..

[205] Ryoo H.D., Gorenc T., Steller H. (2004). Apoptotic Cells Can Induce Compensatory Cell Proliferation through the JNK and the Wingless Signaling Pathways. Dev. Cell.

[206] Lee K., Esselman W.J. (2002). Inhibition of PTPs by H2O2 regulates the activation of distinct MAPK pathways. Free Radic. Biol. Med..

[207] Dunlop E., Tee A. (2014). mTOR and autophagy: A dynamic relationship governed by nutrients and energy. Seminars in Cell & Developmental Biology.

[208] Scherz-Shouval R., Elazar Z. (2007). ROS, mitochondria and the regulation of autophagy. Trends Cell Biol..

[209] Poillet-Perez L., Despouy G., Delage-Mourroux R., Boyer-Guittaut M. (2015). Interplay between ROS and autophagy in cancer cells, from tumor initiation to cancer therapy. Redox Biol..

[210] Son Y.-O., Wang X., Hitron J.A., Zhang Z., Cheng S., Budhraja A., Ding S., Lee J.-C., Shi X. (2011). Cadmium induces autophagy through ROS-dependent activation of the LKB1–AMPK signaling in skin epidermal cells. Toxicol. Appl. Pharmacol..

[211] Boyer-Guittaut M., Poillet L., Liang Q., Bôle-Richard E., Ouyang X., Benavides G.A., Chakrama F.-Z., Fraichard A., Darley-Usmar V.M., Despouy G. (2014). The role of GABARAPL1/GEC1 in autophagic flux and mitochondrial quality control in MDA-MB-436 breast cancer cells. Autophagy.

[212] Cai J., Niu X., Chen Y., Hu Q., Shi G., Wu H., Wang J., Yi J. (2008). Emodin-induced generation of reactive oxygen species inhibits RhoA activation to sensitize gastric carcinoma cells to anoikis. Neoplasia.

[213] Ghavami S., Asoodeh A., Klonisch T., Halayko A.J., Kadkhoda K., Kroczak T.J., Gibson S.B., Booy E.P., Naderi-Manesh H., Los M. (2008). Brevinin-2R1 semi-selectively kills cancer cells by a distinct mechanism, which involves the lysosomal-mitochondrial death pathway. J. Cell. Mol. Med..

[214] Kong Q., Kleinschmidt-DeMasters B.K., Lillehei K.O. (1998). Intralesionally implanted cisplatin plus systemic carmustine for the treatment of brain tumor in rats. J. Surg. Oncol..

[215] Feinendegen L. (2002). Reactive oxygen species in cell responses to toxic agents. Hum. Exp. Toxicol..

[216] Tan S., Sagara Y., Liu Y., Maher P., Schubert D. (1998). The regulation of reactive oxygen species production during programmed cell death. J. Cell Biol..

[217] Pelicano H., Feng L., Zhou Y., Carew J.S., Hileman E.O., Plunkett W., Keating M.J., Huang P. (2003). Inhibition of mitochondrial respiration a novel strategy to enhance drug-induced apoptosis in human leukemia cells by a reactive oxygen species-mediated mechanism. J. Biol. Chem..

[218] Kim J.-W., Tchernyshyov I., Semenza G.L., Dang C.V. (2006). HIF-1-mediated expression of pyruvate dehydrogenase kinase: A metabolic switch required for cellular adaptation to hypoxia. Cell Metab..

[219] Zamzami N., Marchetti P., Castedo M., Decaudin D., Macho A., Hirsch T., Susin S.A., Petit P.X., Mignotte B., Kroemer G. (1995). Sequential reduction of mitochondrial transmembrane potential and generation of reactive oxygen species in early programmed cell death. J. Exp. Med..

[220] Boya P., Gonzalez-Polo R.-A., Poncet D., Andreau K., Vieira H.L., Roumier T., Perfettini J.-L., Kroemer G. (2003). Mitochondrial membrane permeabilization is a critical step of lysosome-initiated apoptosis induced by hydroxychloroquine. Oncogene.

[221] Chomyn A., Attardi G. (2003). MtDNA mutations in aging and apoptosis. Biochem. Biophys. Res. Commun..

[222] Hall M.D., Handley M.D., Gottesman M.M. (2009). Is resistance useless? Multidrug resistance and collateral sensitivity. Trends Pharmacol. Sci..

[223] Pluchino K.M., Hall M.D., Goldsborough A.S., Callaghan R., Gottesman M.M. (2012). Collateral sensitivity as a strategy against cancer multidrug resistance. Drug Resist. Updat..

[224] Wartenberg M., Richter M., Datchev A., Günther S., Milosevic N., Bekhite M.M., Figulla H.R., Aran J.M., Pétriz J., Sauer H. (2010). Glycolytic pyruvate regulates P-Glycoprotein expression in multicellular tumor spheroids via modulation of the intracellular redox state. J. Cell. Biochem..

[225] Pandey V., Chaube B., Bhat M.K. (2011). Hyperglycemia regulates MDR-1, drug accumulation and ROS levels causing increased toxicity of carboplatin and 5-fluorouracil in MCF-7 cells. J. Cell. Biochem..

[226] Bakadlag R. (2016). Collateral Sensitivity of P-glycoprotein Expressing Multidrug Resistant Cells to Tamoxifen is Mediated Through Oxidative Stress. Master of Science Thesis.

[227] Teppo H.-R., Soini Y., Karihtala P. (2017). Reactive oxygen species-mediated mechanisms of action of targeted cancer therapy. Oxid. Med. Cell Longev..

[228] Wan G.-Y., Liu Y., Chen B.-W., Liu Y.-Y., Wang Y.-S., Zhang N. (2016). Recent advances of sonodynamic therapy in cancer treatment. Cancer Biol. Med..

[229] Liu L., He H., Liang R., Yi H., Meng X., Chen Z., Pan H., Ma Y., Cai L. (2018). ROS-inducing micelles sensitize tumor-associated macrophages to TLR3 stimulation for potent immunotherapy. Biomacromolecules.

[230] Brenneisen P., Reichert A.S. (2018). Nanotherapy and Reactive Oxygen Species (ROS) in Cancer: A Novel Perspective. Antioxidants.

[231] Lee H., Shon C., Kim Y., Kim S., Kim G., Kong M.G. (2009). Degradation of adhesion molecules of G361 melanoma cells by a non-thermal atmospheric pressure microplasma. New J. Phys..

[232] Kim G., Kim W., Kim K., Lee J. (2010). DNA damage and mitochondria dysfunction in cell apoptosis induced by nonthermal air plasma. Appl. Phys. Lett..

[233] Watson J. (2013). Oxidants, antioxidants and the current incurability of metastatic cancers. Open Biol..

[234] Yan D., Talbot A., Nourmohammadi N., Sherman J.H., Cheng X., Keidar M. (2015). Toward understanding the selective anticancer capacity of cold atmospheric plasma—A model based on aquaporins. Biointerphases.

[235] Georgescu N., Lupu A.R. (2010). Tumoral and normal cells treatment with high-voltage pulsed cold atmospheric plasma jets. IEEE Trans. Plasma Sci..

[236] Kim J.Y., Ballato J., Foy P., Hawkins T., Wei Y., Li J., Kim S.-O. (2011). Apoptosis of lung carcinoma cells induced by a flexible optical fiber-based cold microplasma. Biosens. Bioelectron..

[237] Ishaq M., Evans M.D., Ostrikov K.K. (2014). Atmospheric pressure gas plasma-induced colorectal cancer cell death is mediated by Nox2–ASK1 apoptosis pathways and oxidative stress is mitigated by Srx–Nrf2 anti-oxidant system. Biochimica et Biophysica Acta (BBA)-Mol. Cell Res..

[238] Kaushik N.K., Kaushik N., Park D., Choi E.H. (2014). Altered antioxidant system stimulates dielectric barrier discharge plasma-induced cell death for solid tumor cell treatment. PLoS ONE.

[239] Volotskova O., Hawley T.S., Stepp M.A., Keidar M. (2012). Targeting the cancer cell cycle by cold atmospheric plasma. Sci. Rep..

[240] Kumar A.P., Loo S.Y., Shin S.W., Tan T.Z., Eng C.B., Singh R., Putti T.C., Ong C.W., Salto-Tellez M. (2014). Manganese superoxide dismutase is a promising target for enhancing chemosensitivity of basal-like breast carcinoma. Antioxid. Redox Signal..

[241] Vaseva A.V., Marchenko N.D., Ji K., Tsirka S.E., Holzmann S., Moll U.M. (2012). p53 opens the mitochondrial permeability transition pore to trigger necrosis. Cell.

[242] Joerger A.C., Fersht A.R. (2008). Structural biology of the tumor suppressor p53. Annu. Rev. Biochem..

[243] Yan N., Shi Y. (2005). Mechanisms of apoptosis through structural biology. Annu. Rev. Cell Dev. Biol..

[244] Yuan S., Yu X., Asara J.M., Heuser J.E., Ludtke S.J., Akey C.W. (2011). The holo-apoptosome: Activation of procaspase-9 and interactions with caspase-3. Structure.

[245] Slee E.A., Harte M.T., Kluck R.M., Wolf B.B., Casiano C.A., Newmeyer D.D., Wang H.-G., Reed J.C., Nicholson D.W., Alnemri E.S. (1999). Ordering the cytochrome c–initiated caspase cascade: Hierarchical activation of caspases-2,-3,-6,-7,-8, and-10 in a caspase-9–dependent manner. J. Cell Biol..

[246] Zou H., Li Y., Liu X., Wang X. (1999). An APAF-1· cytochrome c multimeric complex is a functional apoptosome that activates procaspase-9. J. Biol. Chem..

[247] Ishaq M., Bazaka K., Ostrikov K. (2015). Pro-apoptotic NOXA is implicated in atmospheric-pressure plasma-induced melanoma cell death. J. Phys. D Appl. Phys..

[248] Denicola A., Souza J.M., Radi R. (1998). Diffusion of peroxynitrite across erythrocyte membranes. Proc. Natl. Acad. Sci. USA.

[249] Shi Y. (2002). Mechanisms of caspase activation and inhibition during apoptosis. Mol. Cell.

[250] Kaushik N., Uddin N., Sim G.B., Hong Y.J., Baik K.Y., Kim C.H., Lee S.J., Kaushik N.K., Choi E.H. (2015). Responses of solid tumor cells in DMEM to reactive oxygen species generated by non-thermal plasma and chemically induced ROS systems. Sci. Rep..

[251] Han X., Klas M., Liu Y., Sharon Stack M., Ptasinska S. (2013). DNA damage in oral cancer cells induced by nitrogen atmospheric pressure plasma jets. Appl. Phys. Lett..

[252] Azzariti A., Lacobazzi R.M., Di Fonte R., Porcelli L., Gristina R., Favia P., Fracassi F., Trizio I., Silvestris N., Guida G. (2019). Plasma-activated medium triggers cell death and the presentation of immune activating danger signals in melanoma and pancreatic cancer cells. Sci. Rep..

[253] Smolkova B., Lunova M., Lynnyk A., Uzhytchak M., Churpita O., Jirsa M., Kubinova S., Lunov O., Dejneka A. (2019). Non-Thermal Plasma, as a New Physicochemical Source, to Induce Redox Imbalance and Subsequent Cell Death in Liver Cancer Cell Lines. Cell. Physiol. Biochem..

[254] Santos G.M.P., Oliveira S.C., Monteiro J.C., Fagnani S.R., Sampaio F.P., Correia N.A., Crugeira P.J., Pinheiro A.L.B. (2018). ROS-induced autophagy reduces B16F10 melanoma cell proliferative activity. Lasers Med. Sci..

[255] Filomeni G., De Zio D., Cecconi F. (2015). Oxidative stress and autophagy: The clash between damage and metabolic needs. Cell Death Differ..

[256] Liu Y., Tan S., Zhang H., Kong X., Ding L., Shen J., Lan Y., Cheng C., Zhu T., Xia W. (2017). Selective effects of non-thermal atmospheric plasma on triple-negative breast normal and carcinoma cells through different cell signaling pathways. Sci. Rep..

[257] Kong A.-N.T., Yu R., Chen C., Mandlekar S., Primiano T. (2000). Signal transduction events elicited by natural products: Role of MAPK and caspase pathways in homeostatic response and induction of apoptosis. Arch. Pharm. Res..

[258] Henry Y., Ducrocq C., Drapier J.-C., Servent D., Pellat C., Guissani A. (1991). Nitric oxide, a biological effector. Eur. Biophys. J..

[259] Liedtke K.R., Bekeschus S., Kaeding A., Hackbarth C., Kuehn J.-P., Heidecke C.-D., von Bernstorff W., von Woedtke T., Partecke L.I. (2017). Non-thermal plasma-treated solution demonstrates antitumor activity against pancreatic cancer cells in vitro and in vivo. Sci. Rep..

[260] Li W., Yu K.N., Ma J., Shen J., Cheng C., Zhou F., Cai Z., Han W. (2017). Non-thermal plasma induces mitochondria-mediated apoptotic signaling pathway via ROS generation in HeLa cells. Arch. Biochem. Biophys..

[261] Kang S.U., Cho J.H., Chang J.W., Shin Y.S., Kim K.I., Park J.K., Yang S.S., Lee J.S., Moon E., Lee K. (2014). Nonthermal plasma induces head and neck cancer cell death: The potential involvement of mitogen-activated protein kinase-dependent mitochondrial reactive oxygen species. Cell Death Dis..

[262] Chen Z., Lin L., Cheng X., Gjika E., Keidar M. (2016). Treatment of gastric cancer cells with nonthermal atmospheric plasma generated in water. Biointerphases.

[263] Nguyen N.H., Park H.J., Yang S.S., Choi K.S., Lee J.-S. (2016). Anti-cancer efficacy of nonthermal plasma dissolved in a liquid, liquid plasma in heterogeneous cancer cells. Sci. Rep..

[264] Karki S.B., Gupta T.T., Yildirim-Ayan E., Eisenmann K.M., Ayan H. (2017). Investigation of non-thermal plasma effects on lung cancer cells within 3D collagen matrices. J. Phys. D Appl. Phys..

[265] Kumar N., Attri P., Choi E.H., Uhm H.S. (2015). Influence of water vapour with non-thermal plasma jet on the apoptosis of SK-BR-3 breast cancer cells. RSC Adv..

[266] Choi J.-S., Kim J., Hong Y.-J., Bae W.-Y., Choi E.H., Jeong J.-W., Park H.-K. (2017). Evaluation of non-thermal plasma-induced anticancer effects on human colon cancer cells. Biomed. Opt. Express.

[267] Kaushik N.K., Kim Y.H., Han Y.G., Choi E.H. (2013). Effect of jet plasma on T98G human brain cancer cells. Curr. Appl. Phys..

[268] Conway G.E., Casey A., Milosavljevic V., Liu Y., Howe O., Cullen P.J., Curtin J.F. (2016). Non-thermal atmospheric plasma induces ROS-independent cell death in U373MG glioma cells and augments the cytotoxicity of temozolomide. Br. J. Cancer.

[269] Schmidt A., Rödder K., Hasse S., Masur K., Toups L., Lillig C.H., von Woedtke T., Wende K., Bekeschus S. (2016). Redox-regulation of activator protein 1 family members in blood cancer cell lines exposed to cold physical plasma-treated medium. Plasma Process. Polym..

[270] Kaushik N., Kumar N., Kim C.H., Kaushik N.K., Choi E.H. (2014). Dielectric barrier discharge plasma efficiently delivers an apoptotic response in human monocytic lymphoma. Plasma Process. Polym..

[271] Coates A., Abraham S., Kaye S.B., Sowerbutts T., Frewin C., Fox R., Tattersall M. (1983). On the receiving end—Patient perception of the side-effects of cancer chemotherapy. Eur. J. Cancer Clin. Oncol..

[272] Pedersen B., Koktved D.P., Nielsen L.L. (2013). Living with side effects from cancer treatment—A challenge to target information. Scand. J. Caring Sci..

[273] Peng X., Gandhi V. (2012). ROS-activated anticancer prodrugs: A new strategy for tumor-specific damage. Ther. Deliv..

[274] Rajivgandhi G., Maruthupandy M., Quero F., Li W.J. (2019). Graphene/nickel oxide nanocomposites against isolated ESBL producing bacteria and A549 cancer cells. Mater. Sci. Eng. C.

[275] NavaneethaKrishnan S., Rosales J.L., Lee K.-Y. (2019). ROS-Mediated Cancer Cell Killing through Dietary Phytochemicals. Oxid. Med. Cell. Longev..

[276] Zhou Z., Song J., Nie L., Chen X. (2016). Reactive oxygen species generating systems meeting challenges of photodynamic cancer therapy. Chem. Soc. Rev..

